# Home Alone: Widows’ Well-Being and Time

**DOI:** 10.1007/s10902-023-00622-w

**Published:** 2023-01-20

**Authors:** Maja Adena, Daniel Hamermesh, Michał Myck, Monika Oczkowska

**Affiliations:** 1 Wissenschaftszentrum Berlin (WZB), 10785 Berlin, Germany; 2 University of Texas at Austin, Austin, TX 78712 USA; 3 Institute for the Study of Labor, 53113 Bonn, Germany; 4 National Bureau of Economic Research, Cambridge, USA; 5 Centre for Economic Analysis, Cyfrowa 2, 71441 Szczecin, Poland; 6 University of Greifswald, 17489 Greifswald, Germany

**Keywords:** Widowhood, Well-being, Social networks, Time use, I31, I19, J14

## Abstract

Using data from the Survey of Health, Ageing and Retirement in Europe (SHARE, 2004–17) and time diaries from Poland (2013), the U.S. (2006–16), the U.K. (2014–15) and France (2009–10), we examine differences between widowed and partnered older women in well-being and its development in widowhood. Most importantly, our analysis accounts for time use, an aspect which has not been studied previously. We trace the evolution of well-being of women who become widowed by comparing them with their matched non-widowed ‘statistical twins’ and examine the role of an exceptionally broad set of potential moderators of widowhood’s impact on well-being. We confirm a dramatic decrease in mental health and life satisfaction after the loss of partner, followed by a slow partial recovery over a 5-year period. An extensive set of controls recorded prior to widowhood, including detailed family ties and social networks, provides little help in explaining the deterioration in well-being. Unique data from time-diaries kept by older women in several European countries and the U.S. tell us why: the key factor behind widows’ reduced well-being is increased time spent alone.

**Supplementary Information:**

The online version contains supplementary material available at 10.1007/s10902-023-00622-w.

## Introduction

Widowhood is the fate of most married women surviving past age 70 in the majority of wealthy countries (U.S. American Community Surveys 2006–17, and United Nations 2008), and in many countries the number of women who become widows has grown rapidly over the last decade. For example, in Denmark, Greece, Italy, Portugal, and Switzerland the number of deceased married men aged 70+ increased by over 15% between 2007 and 2019, while in Austria, Czech Republic and the Netherlands it grew by more than 20% (see Figure S1 and Table S1 in the online Supplementary Information). These trends reflect both increased longevity and the coming of age of subsequent, more numerous cohorts, but the numbers leave no doubt that widowhood has become a challenge for an increasing number of women. Given the years of life lost to COVID-19, both the incidence of widowhood and the average lifetime in widowhood will grow in the coming years.

In this article we study the conditions which could help explain the difference in well-being between widowed and non-widowed older women. We combine the advantages of several datasets to address the evolution of well-being before and after the death of a partner. We focus only on widowed women because of the size and relevance of this group, and because, unlike among older widowed men, opportunities for finding a new partner are very scarce. We apply advanced matching methods to provide a comprehensive picture of the evolution of well-being before and after losing a partner. Unique survey modules such as *end-of-life* interviews, social network information, and life histories allow us to study the role of important conditions which may influence well-being of widows. We document differences in well-being between widowed and non-widowed ‘statistical twins,’ which persist even 5 years after the spouse’s death, with recovery being slower among women whose partners died suddenly. We show that the drop in well-being around widowhood is independent of a large set of characteristics and cannot be explained by variation in physical closeness of family or a broader social network. We complement the longitudinal analysis with detailed multivariate analyses linking time use to life satisfaction using cross-section data from four quite different economies, Poland, the United States, the United Kingdom, and France. We examine differences in daily time use between widowed and married individuals and demonstrate that the key aspect to understanding dynamics of well-being in widowhood is time spent alone. This points towards potential areas of support and suggestions both for the widows’ families and possibly for public policy. Reducing widows’ time spent alone could result in major improvements in their quality of life.

This article is novel in several ways. No earlier study that addressed developments in life satisfaction around widowhood has been as extensive as ours. To our knowledge this is the first paper to analyze the evolution of well-being before and after spousal loss through detailed advanced matching methods using an extensive array of individual-level, unique information. More generally, it is the first to study the link between explicit measures of people’s use of time and their happiness in the context of examining a major life-cycle event. As such, it has implications for how to analyze the impact of other crucial life-cycle events, such as birth or marriage, on mental health and life satisfaction or happiness. Despite the vast literature on widowhood, studies involving time use in this context are sparse. Ours is the first to examine widows’ time use in enough detail to allow an understanding of what older women do, and with whom they do it, in relation to their life satisfaction. Given the importance of this demographic group, the analysis thus can illuminate the unique problems widows face in their daily lives.

## Literature Review

We move in this review from the general issues of happiness and of time use to specifics dealing with widowhood, and then to those studies that have examined interactions between two of these. We then delve into the longitudinal aspects and finish with a discussion of potential moderators.

The novelty of our work is its introduction of time use—based upon actual diaries of what people do rather than general recollections—and life satisfaction, with a specific focus on widowhood. Given the vast literature on happiness, we only point to a few important recent contributions including Clark et al. (2008), Clark and Georgellis (2013), Layard (2006), Lucas et al. (2003), Luhmann et al. (2012), and the helpful survey by Diener et al. (2018). The scales that we use to analyze life satisfaction are discussed in various of these studies.

Researchers have analyzed various topics with time-use data, going far beyond economists' focus on work for pay, which constitutes less than 20% of the average adult's day and is only the second most common use of time (sleeping being first). This analysis was stimulated by the remarkably perceptive work of Reid (1934) which has come to be recognized both as the underpinning for the theoretical analysis of people’s use of their time and for subsequent empirical studies. An early discussion of time-diary data is provided by Juster and Stafford (1991), with a nice example of their use in Gershuny (2003). Hamermesh (2019) presented a large review of time-use studies and their implications for discussing a variety of differences by demographics and country.

While the number of studies of widows’ behavior seems substantial, it falls short of this group’s importance in today’s societies. Avis et al. (1991) presented a general discussion, looking at women who, in a prospective study, lost their husbands and comparing them to those continuously married. Emphasizing the importance of controlling for pre-widowhood characteristics, they provide some insights into the detrimental impact of widowhood on mental health and income, noting at the same time no effect on their physical health. In a systematic review, Stahl and Schulz (2014) specifically examined widows’ health-related behavior, documenting consistent evidence of weight loss and worsened nutrition patterns, and some evidence pointing towards impaired sleep quality and an increase of alcohol consumption after widowhood. In a broader review of the consequences of bereavement, Stroebe et al. (2007) considered health outcomes such as excess mortality and elevated risk of ill physical health directly. While Holm et al. (2019) provided similar evidence on this topic, in their review they emphasized the role of social support when suffering from emotional distress and poor health following widowhood. Moon et al. (2011) further examined the ‘widowhood effect’—systematizing earlier contributions related to the higher incidence of mortality after losing a partner. Posner (1997) proposed an interesting policy change involving the reallocation of monetary expenditures on healthcare from older women to older men based on comparisons of gender differences in older persons’ health and longevity. This idea was tested on German panel data by Wunder and Schwarze (2014), who find that the detrimental effect of a partner’s death on life satisfaction is temporary, and thus conclude that Posner’s policy suggestion would not be beneficial for widows.

There has been a substantial focus in the literature specifically on widows’ mental health. General examinations of widows’ mental health in response to bereavement are by Spahni et al. (2015) and Siflinger (2017). The first study examines the role of three factors in the process of adaptation to loss: trait resilience, marital history and circumstances accompanying death, with the first being key. On top of the adaptation effects, the latter study investigates the anticipation effects for the probability of depression attributed to caregiver burden. Choi et al. (2013) have used the SHARE data in cross-section analyses comparing depression among widows and others in Europe, an analysis similar to that of Richardson et al. (2020). Li et al. (2005) studied how Chinese widows’ depression is affected by the extent of their social ties, documenting that while adult children’s support cushioned the negative effect of widowhood on mental health, spousal support received in years preceding death enhanced vulnerability afterwards. This analysis and the findings were mirrored for Korea by Jeon et al. (2013). In the context of more broadly defined social interactions, when comparing neighborhoods in the United States with different concentrations of widowed individuals, Subramanian et al. (2008) suggested the potentially beneficial impact of the opportunity of interacting with other widow(er)s for their survival. Kristiansen et al. (2019) summarized the literature on depression in widowhood. The general conclusion from this literature is the unsurprising one that widows tend to be more depressed than nonwidowed older women.

The literature examining life satisfaction in widowhood is much sparser. The majority of studies, including Bennett and Soulsby (2012), Bratt et al. (2017), Cheng et al. (2014), and Steptoe et al. (2015), did not present the problem in a satisfactorily far-reaching longitudinal setting. Even when they did, as in Nakagawa and Hülür (2021), those analyses are based on relatively small samples and/or do not account for what might be crucial explanatory variables, e.g. partner’s sociodemographic information or health and health related behavior before his death. Infurna et al. (2017) and Wünsche et al. (2020) are two notable exceptions, both having looked at trajectories of life satisfaction around widowhood based on the German Socio-Economic Panel data. Both studies observe some deterioration in life satisfaction before the partner’s death, except when the death was unanticipated. A reduction in well-being already before the partner’s death is considered an implication of anxiety and stress related to the worsening health of the partner and to the burden of caring responsibilities (Gerlich & Wolbring, 2021; Schulz et al., 2001), mentioned already above in the context of mental health (Siflinger, 2017).

Substantial differences in the implications for well-being between anticipated and unanticipated deaths have been well documented also for the period following the partner’s death. Kristensen et al. (2012) and Scott et al. (2020) pointed towards serious implications for mental health after deaths occurring suddenly, caused by accidents, natural disasters, or short-term illnesses. Through an extensive literature review, the former study provided substantial evidence that sudden deaths lead to a much more elevated risk of mental health disorders and can be associated with slower recovery.

It is important to note that studies focused on factors that differentiate the effects of widowhood rarely look at the differences in trajectories of certain outcomes, instead reporting level differences. One exception again is the analysis by Infurna et al. (2017), who documented distinct trajectories depending on the individual’s age, health, disability, and social participation prior to widowhood. According to their findings based on a representative sample of the German population, younger individuals in better health and more socially active adapt more easily to spousal loss. On the contrary, studies by Archer (1998) and Hansson and Stroebe (2007) reported stronger negative effects of widowhood at younger ages. Education was documented to have either no (Stevens, 1995) or a positive effect (Elwell & Maltbie-Crannell, 1981) on the process of recovery after spousal death, while economic resources seemed not to play much of a role (Hansson & Stroebe, 2007; Kung, 2020; Martikainen & Valkonen, 1998). Implications of the type of residence have so far received little consideration. Fengler and Danigelis (1982) reported that widows living in urban settings have lower levels of life satisfaction and subjectively consider themselves as more disadvantaged.

A distinct strand of the literature is devoted to gender differences in widowhood, indicating important disparities between widows and widowers regardless of the outcome analyzed. Widowers’ life satisfaction trajectories following partner’s death differ from those of widows’, with bereaved men reporting lower levels of life satisfaction as compared to women (Bratt et al., 2017). Other studies showed important differences in other outcomes (depression, physical health, morbidity), although with inconclusive results about whether the consequences of spousal loss are more harmful for widows or for widowers. Taking the example of the impact of widowhood on mental health, some studies report that the effect of widowhood is more adverse for men (Lee et al., 2001), while in other settings women are indicated as the group more likely to develop depression as a result of bereavement (Chen et al., 2020; Thompson et al., 1989). An interesting summary of the literature related to the gendered nature of widowhood is presented by Carr and Bodnar-Deren (2009), who point out that gender differences observed in the aftermath of bereavement come down to different sources of vulnerability for men and for women. As the main ones, the authors list reduction in social support and health protection for the first group and financial strain for the latter.

A few studies have analyzed what widows do during their widowhood, most using cross-section data, most with small samples, none with time-diary data and none examining well-being in this context (Hahn et al., 2011, 2014; Utz et al., 2004). Hamermesh et al. (2022) used large time-diary samples from several countries to see how widows’ time use differs from that of other women who are similar along a large variety of demographic characteristics. They found that in comparison to married women, widows engage less in home production, and they are alone most of the time that they had previously spent with their husbands, although they observe a slight increase in time spent with friends and family shortly after partner’s death. But that study did not examine any issues of mental health or well-being.

A few studies have examined loneliness based on comparing expressions of loneliness. Yang and Gu (2021) prove that feelings of loneliness in widowhood are very persistent. While the likelihood of loneliness decreases with the duration of widowhood, the probability of feeling lonely is significantly higher for widowed individuals compared to couples even after a remarkably long time since partner’s death. On a sample of older people generally, and without focusing on widows, Steptoe et al. (2013) conclude that both loneliness and social isolation negatively impact mortality, but the first one cannot be considered as the channel through which social isolation affects health. Utz et al. (2014) also address the important distinction between being alone and feeling lonely, and they focus specifically on bereaved individuals. Although they confirm an overlap between social support and feeling of loneliness and find that receiving more social support is associated with lower levels of loneliness, they argue that loneliness in widowhood cannot be tackled solely with interventions designed to increase social support. While Utz et al. (2014) look into the consequences of being alone following spousal loss, neither this study nor any other (to our knowledge) adds time use per se to the mix.

Widowhood is considered an important life-changing event, and as such its implications can be compared to the impact of other shocks on well-being. This issue has already been addressed in the literature. Clark et al. (2008) and Clark and Georgellis (2013) analyzed the patterns of anticipation and adaptation to widowhood, but also offered a comparison to other life events like marriage, divorce or birth of a child, and labor market events such as episodes of unemployment or layoffs. Stallings et al. (1997) took a closer look at changes in well-being after death of children/parents, health decline, hospitalization, and retirement.

Our research brings together various strands of the literature discussed here, weaving the analysis of life satisfaction, depression, time use, and loneliness into the same discussion, and doing so specifically in the context of comparing widows to otherwise identical married women. Moreover, much of that interweaving is based on longitudinal data, thus accounting for possible unobservable differences between observably similar widows and married women. Finally, we analyze the role of loneliness in affecting life satisfaction explicitly by looking at who widows spend time with (or whether they are alone). With this we complement the existing literature which has focused rather on expressions of feelings of loneliness.

The main novelties are the examination of comprehensive, generally representative time-use data and of representative longitudinal data that allow examining the dynamics of the adjustment of life satisfaction to widowhood and the role of time use in affecting it. Regrettably, because there are no longitudinal datasets combining information on time use and life satisfaction among the same people, the “holy grail”—the dynamic analysis of time use and satisfaction together—cannot be attained.

## Data and Methods

This study draws on data from a well-established representative panel survey—the Survey of Health, Ageing and Retirement in Europe (SHARE), which is supplemented with time-use surveys collected in France, Poland, the U.K., and the U.S.

### Sample Selection and Matching Procedure Employed in the Analysis of the SHARE

SHARE is an international and multidimensional panel study of individuals aged 50 and over, carried out regularly in Europe and Israel since 2004 (Börsch-Supan, 2020; Börsch-Supan et al., 2013). Importantly from the perspective of the analysis of widowhood, apart from the regular interviews capturing the current living situation of participants, SHARE collects information about the final months preceding the death of respondents who had participated in the survey at least once beforehand (the so-called *end-of-life* interviews; Orlovic et al., 2017). SHARE also collects *SHARE-Life* interviews focusing on retrospective life histories of participants. These retrospective interviews were conducted in wave 3 of SHARE and, for respondents who joined SHARE later, again in wave 7. Thus, in wave 7 two types of interviews were conducted–regular interviews for panel members from before wave 3, and *SHARE-Life* interviews for respondents who first participated in wave 4 or later.^1^

The sample used here consists of women who participated in at least two waves of the survey, between waves 1 (conducted in years 2004–05) and 7 (2017), excluding wave 3, which collected only life history information and thus does not contain questions on the outcomes we examine. We refer to two waves of the survey on which we condition the sample as ‘principal’ waves. They are defined as: (1) for the widowed sample the ‘principal’ waves are two instances of consecutive participation in the survey separated by the death of the partner. These women lived with their partners in the first of the ‘principal’ waves. The partner is required to have participated at least once, either in the first of the ‘principal’ waves or earlier. We refer to the *end-of-life* interview for additional information on the circumstances of the partner’s death. (2) For women living in couples in both ‘principal’ waves we require that the partner also participated in another SHARE interview, either at the time of the second ‘principal’ wave or in a later wave.

**Fig. 1:**
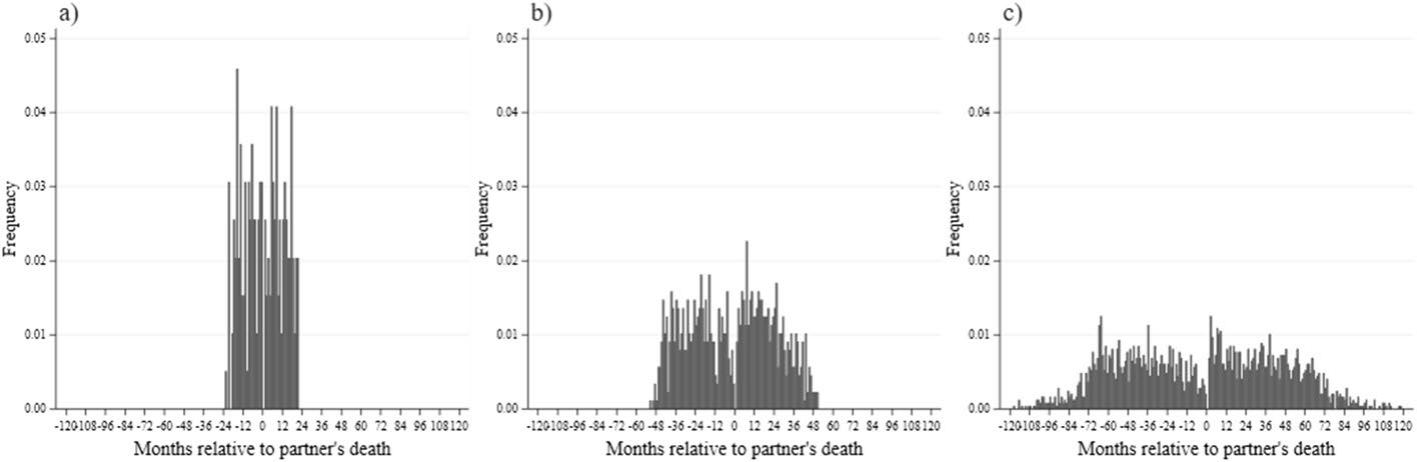
Survey participation and interview timing relative to time of partner’s death (SHARE). *Source*: own calculations based on SHARE data. *Note*: Number of months between the first and the last interview in the survey (sample used for the analysis of life satisfaction): **a** 0–24 months: 98 observations. **b** 25–60 months: 441 observations. **c** More than 60 months: 2537 observations. Negative values represent time before the death of partner; positive values—the months after the death of partner

The sample used includes only individuals who provided information on early childhood characteristics collected over the course of their participation. This information is used in the matching procedure needed to construct the control (non-widowed) sample. Since eligibility for the SHARE interview is not based on formal marital status but rather on living with a partner in the same household, we do not differentiate between married and cohabiting couples. Furthermore, apart from the two ‘principal’ waves, we include information from all other waves in which the widow was interviewed, and the same principle is applied to the control sample. This allows us to examine the evolution of the outcomes far into the past before the partner’s death and far into the years following it. In Fig. 1a–c we present for the final sample the distribution of the time span between the very first and very last interview for each widow with respect to the timing of their partners’ death. The data in Fig. 1a–c are split into three groups—those who have been in the survey for less than 2 years (98 widows in the sample), those who participated in the panel between 25 and 60 months (441) and—the largest group—those who have been followed in the survey for more than 60 months (2537).^2^ For all three groups there is a discontinuity in the number of months between the partner’s death (time 0 in Fig. 1) and the time of the interview at around the time of his death—a clear and understandable consequence of non-participation in weeks immediately prior and right after the time of passing of the partner. Except for this discontinuity, however, the timing of death is fairly evenly distributed with respect to the pattern of survey participation.

In wave 7 of SHARE some of the participants received the regular panel questionnaire, while others, who did not participate in wave 3, were interviewed on their retrospective life history (*SHARE-Life* interview). The latter sample was additionally asked a subset of the regular questions, including the question on life satisfaction, but excluding questions used to construct measures of mental health. This results in small differences in the number of observations depending on the outcome being studied.

Although partners’ death can as such be treated as exogenous with respect to the examined outcomes, in the sense that wives’ well-being is unlikely to be a determining factor of their partners’ death, matching the widowed to a control sample is still necessary from the point of view of the causal interpretation of our results. A bias would result, for example, if women whose partners died early were also characterized by overall lower levels of well-being (before and after widowhood).

The process of matching is done in two stages. The first, exact stage is done by country. Within these country cells individuals are matched based on the propensity score for becoming a widow. In this second stage, we account for socio-demographic information (age, country, education, characteristics of the place of living, number of living children and grandchildren), for health status in childhood as well as for the pattern of survey participation. Additionally, since widowhood is strongly related to the characteristics of the partner prior to his death, we also control for the partner’s age, education, self-reported health, and BMI category at the initial wave of observation, years of smoking, having siblings, and the age of his father (at death or at the time of the initial wave if the father was still alive).^3^ We apply nearest neighbor propensity score matching with replacement within each country cell, conditional on common support.^4^ Table 1 provides basic information on the number of widows by country, splitting the samples additionally into those whose partners died suddenly (as a result of an accident or following an illness which lasted less than a month) and those whose partners’ illness lasted more than a month. Our results show that the processes of adjusting to loss following a ‘sudden’ and ‘non-sudden’ death of a partner are significantly different. This distinction is informative and seems of particular importance currently, at the time when many older people are losing their partners due to an unexpected death from COVID-19.

**Table 1:** Final matched sample of widows (SHARE). *Source*: own calculations based on SHARE data

Country	Sample for mental health analysis				Sample for life satisfaction analysis			
	Number of widows in sample		Average time (months) between partner’s death and...		Number of widows in sample		Average time (months) between partner’s death and…	
	Non-sudden	Sudden	…initial obs	…final obs	Non-sudden	Sudden	…initial obs	…final obs
Austria	81	26	51.44	50.93	113	33	53.04	40.31
Belgium	147	35	62.22	56.38	162	36	61.35	51.61
Croatia	…	…	…	…	16	13	8.69	11.79
Czech Republic	139	54	40.77	45.81	179	68	42.13	39.86
Denmark	105	28	63.77	53.02	122	33	61.33	47.92
Estonia	139	55	28.71	42.45	222	89	41.25	31.51
France	126	30	62.04	55.73	152	33	61.75	47.39
Germany	81	27	60.30	49.18	114	34	55.41	39.06
Greece	80	67	86.47	53.87	101	77	79.44	48.51
Israel	92	21	56.48	62.34	119	21	63.46	52.74
Italy	171	57	72.75	48.91	197	63	68.91	45.94
Luxembourg	4	1	11.20	27.20	18	2	20.55	19.50
Netherlands	72	27	45.75	48.63	72	27	45.94	48.42
Poland	105	36	58.91	52.07	107	37	59.19	51.35
Portugal	17	5	26.64	55.23	43	10	46.66	36.36
Slovenia	29	7	23.89	39.36	81	18	35.49	24.03
Spain	215	68	60.64	51.42	303	88	57.39	44.91
Sweden	134	26	70.12	64.77	153	28	66.96	61.65
Switzerland	54	20	59.11	46.62	66	26	58.65	38.79
Total	1791	590	57.84	51.76	2340	736	56.20	43.95

Since in wave 7 mental health outcomes were not collected from all participants, we use different samples of widows for the analyses focused on these outcomes and for the analyses focused on life satisfaction. For each of these samples we also conduct a separate matching procedure using different gross samples of partnered women, as described in the text. Countries in the table are ordered alphabetically. Two other countries collected data in at least two waves of SHARE but were not taken into account in the analysis: Hungary and Ireland. In Hungary data was collected in waves 4 and 7, however due to the long interval between them a relatively small proportion of respondents from wave 4 participated in the latter wave. Ireland participated only in waves 2 and 3, and in the latter collected only life history information

After the treatment and control samples are matched, we impute the timing of the ‘placebo’ deaths for non-widowed individuals to trace the path of outcomes with reference to the timing of this placebo treatment. We assign it so that the time between the ‘principal’ wave prior to and the second ‘principal’ wave after this placebo treatment is proportionally the same with respect to the actual death of her partner as for the matched widowed individual.

In Table 2 we present a selection of descriptive statistics for the matched sample of widowed and married women, separately for the samples used in the analysis of mental health outcomes and life satisfaction (full details given in Table S3 in the Supplementary Information). The widowed and married samples are well balanced in terms of the basic sociodemographic characteristics. The mean age of women in both samples (at the time of the initial observation) is about 70 years old and their husbands are about 4 years older. The majority of couples (about 55%) live in small towns and villages, very few have no children (only about 5%), and 40% have three children or more. Apart from the basic descriptive statistics on the variables used for the matching exercise, Table 2 also provides some details on our outcome variables—as measured in the final interview before the death of partner (actual, or imputed in the control group). The average values for the dimensions of well-being among widows before their partner’s death are very similar to those of non-widowed women.

**Table 2:** Descriptive statistics of final matched samples for the analysis of mental health outcomes and life satisfaction (SHARE). *Source*: own calculations based on SHARE data

	Sample for mental health analysis (Fig. 2a–d)				Sample for life satisfaction analysis (Fig. 3a–d)			
	Control n = 2381		Widowed n = 2381		Control n = 3076		Widowed n = 3076	
*Demographic information*								
Widows due to *sudden* death (%)	–	–	590	(24.8%)	–	–	736	(23.9%)
Mean age at initial wave (SD)	69.84	(8.73)	70.19	(8.51)	70.31	(8.48)	70.56	(8.54)
Education (%)								
Primary or less, other	1024	(43.0%)	1016	(42.7%)	1262	(41.0%)	1267	(41.2%)
Lower secondary	375	(15.8%)	424	(17.8%)	506	(16.5%)	548	(17.8%)
Higher	982	(41.2%)	941	(39.5%)	1308	(42.5%)	1261	(41.0%)
Area of living in initial wave (%)								
A big city, suburbs or a large town	1073	(45.1%)	1069	(44.9%)	1303	(42.4%)	1339	(43.5%)
A small town or rural	1301	(54.6%)	1302	(54.7%)	1763	(57.3%)	1724	(56.1%)
Missing	7	(0.3%)	10	(0.4%)	10	(0.3%)	13	(0.4%)
Number of children alive in initial wave (%)								
No children	114	(4.8%)	114	(4.8%)	168	(5.5%)	154	(5.0%)
1 child	431	(18.1%)	407	(17.1%)	541	(17.6%)	549	(17.9%)
2 children	862	(36.2%)	932	(39.1%)	1142	(37.1%)	1213	(39.4%)
3+ children	974	(40.9%)	928	(39.0%)	1225	(39.8%)	1160	(37.7%)
Distance and frequency of contact with children in initial wave (%)								
Close child*	1131	(47.5%)	1119	(47.0%)	1400	(45.5%)	1467	(47.7%)
No close child*	1224	(51.4%)	1243	(52.2%)	1638	(53.3%)	1583	(51.4%)
Missing	26	(1.1%)	19	(0.8%)	38	(1.2%)	26	(0.9%)
Mean partner's age in initial wave (SD)	73.77	(8.84)	74.04	(8.69)	74.27	(8.80)	74.42	(8.74)
*Well-being outcomes in the last interview before partner’s death*								
In the last month “cried at all” (%)	881	(37.0%)	947	(39.8%)	–	–	–	–
In the last month “felt that would rather be dead” (%)	192	(8.1%)	200	(8.4%)	–	–	–	–
Mean number of symptoms on 0–12 EURO-D scale (SD)	3.18	(2.47)	3.22	(2.54)	–	–	–	–
Depression risk (4+ symptoms, %)	920	(38.6%)	896	(37.6%)	–	–	–	–
Mean life satisfaction on 10–0 scale (SD)	–	–	–	–	7.49	(1.80)	7.23	(1.99)

See Table 1. *Close child—one or more children living in same household/building or less than 1 km away, contacts at least once a week; No close child—no children or children living further away and less frequent contact

These outcomes are examined in detail in the final step of the analysis where we look at their evolution over time with respect to the timing of the actual or imputed (hypothetical) time of death of the partner (marked as time 0 in the figures). To allow for a flexible specification, we use a local polynomial regression and fit it against the number of months before and after the partner’s death (actual or imputed; $m_{i,t}$). The specification thus takes the form:

$$y_{i,t} = g\left( {m_{i,t} } \right) + v_{i} + \varepsilon_{i,t} ,$$

where $g\left( . \right)$ is the local polynomial function fitted separately for (1) the non-widowed sample, (2) the widowed sample before partner’s death, and (3) the widowed sample after partner’s death. Under the conditional independence assumption there should be no systematic differences in $v_{i}$ between the widowed and non-widowed samples. The estimates are produced using the STATA built-in *lpoly* command with Epanechnikov kernel-weighted local polynomial smoothing at 60 points with a 0.9 bandwidth. Robustness tests using different numbers of smoothing points and bandwidths in the proximity of these values produce very similar results.

SHARE includes a number of unique modules that were collected in selected waves of the survey. One provides very detailed information on respondents’ social networks—individuals with whom they “often discuss things that are important” (type of relationship, frequency of contact, how close one feels to this person). To be able to use detailed information on social networks, we further focus on the sample of women living in a couple at the time of wave 4 of the SHARE survey (when this module was implemented) with a partner who was observed at the same time (or during an earlier wave, provided the partner was still the same at the time of wave 4). From among these respondents, we look at the outcomes of those who were re-interviewed in wave 6 and who were 70 years old or older at that time, with some of them becoming widowed after the wave 4 interview. The non-widowed women in the estimation sample include those who continued to live with the same partner (and their partner was interviewed at the time of wave 6 or later). Table 3 provides descriptive statistics for this sub-sample, giving an overview of the distribution of outcomes in the regressions.

**Table 3:** Descriptive statistics of the sub-sample in the social network analysis (SHARE). *Source*: own calculations based on SHARE data

	n = 3056	
Marital status in latter wave (%)		
Widowed	316	(10.3%)
Other	2740	(89.7%)
Life satisfaction (10–0 scale) in latter wave (%)		
Satisfied (8–10 points)	1984	(64.9%)
Not satisfied (0–7 points)	1072	(35.1%)
Loneliness in latter wave (%)		
Feeling lonely often or some of the time	785	(25.7%)
Feeling lonely hardly ever or never	2271	(74.3%)
Mean age in latter wave (SD)	76.15	(4.58)
Education (%)		
Primary or less	1026	(33.6%)
Secondary and post-secondary non-tertiary	1504	(49.2%)
Tertiary	493	(16.1%)
Other	33	(1.1%)
Area of living in initial wave (%)		
A big city	436	(14.3%)
The suburbs or outskirts of a big city	274	(9.0%)
A large town	476	(15.6%)
A small town	782	(25.6%)
A rural area or village	1088	(35.6%)
House ownership in initial wave (%)		
Owner	2493	(81.6%)
Member of a cooperative	57	(1.9%)
Tenant or subtenant	279	(9.1%)
Rent free	227	(7.4%)
Self-reported health in initial wave (%)		
Excellent	121	(4.0%)
Very good	386	(12.6%)
Good	1102	(36.1%)
Fair	1064	(34.8%)
Poor	383	(12.5%)
Number of children alive in initial wave (%)		
No children	138	(4.5%)
1 child	503	(16.5%)
2 children	1272	(41.6%)
3+ children	1143	(37.4%)
Distance and freq. of contact with child. in initial wave (%)		
No children	138	(4.5%)
Same household	467	(15.3%)
Same building or less than 1 km and contact every day	619	(20.3%)
Between 1–5 km and contact every day	213	(7.0%)
Less than 5 km and contact less often	640	(20.9%)
Further than 5 km and contact every day	229	(7.5%)
Further than 5 km and contact more than once a week	418	(13.7%)
Further than 5 km and contact less often	332	(10.9%)
Size of the social network in initial wave (%)		
Empty	44	(1.4%)
1–7 people	3012	(98.6%)
Partner with whom one feels close named in social network in initial wave (%)		
Yes	2128	(69.6%)
No	928	(30.4%)
Child with whom one feels close named in social network in initial wave (%)		
Yes	1695	(55.5%)
No	1361	(44.5%)
Friend with whom one feels close named in social network in initial wave (%)		
Yes	618	(20.2%)
No	2438	(79.8%)
Satisfaction with social network (10–0 scale) in initial wave (%)		
Satisfied (10 points)	1422	(46.5%)
Not satisfied (0–9 points)	1634	(53.5%)

### Sample Selection and Descriptive Statistics in the Time Use Surveys

The time-use surveys measure the amount of time people spend doing various activities during a day, including information on who they spend time with. Respondents complete a detailed written diary of their activities on the previous day. Such diaries are an effective means of capturing rich data on how people spend their time, their location throughout the day, and who they spend their time with (Juster & Stafford, 1991). We aggregate reported activities into five categories: home production, activities that others could perform for you (Reid, 1934); sleep; other personal activities; TV-watching; and other leisure (Hamermesh, 2019). Follow-up interviews provide additional demographic, economic, and social information about households and individuals. We use four time-use surveys, conducted in France, French Time Use Survey *Emploi du Temps* 2009–2010 (French Data Archives for Social Sciences ADISP, 2010); Poland, Polish Time Use Survey *Budżet Czasu Ludności* 2013 (Polish Central Statistical Office, 2013); the U.K., United Kingdom Time Use Survey UKTUS 2014–2015 (Gershuny, 2003; Gershuny & Sullivan, 2017), and the U.S., American Time Use Survey ATUS 2006–2008, 2010–2014, 2016 (Hofferth et al., 2020).^5^

Table 4 summarizes general information on differences in time use between married and widowed individuals in each country using information for all women aged 70+. The sub-sample used in our analysis was restricted to those women who also provided details on other variables used in the regressions, most importantly—life satisfaction. Table 5 presents descriptive statistics for these sub-samples in each country. Due to the questionnaire design, and because some questions were only asked of sub-samples in France, the U.K., and the U.S., the sub-samples used for our analysis are much smaller than the full samples in Table 4.

**Table 4:** Time use: widows compared to married women. *Source*: own calculations based on American, French, Polish and United Kingdom Time Use Survey data

	Polish Time Use Survey		American Time Use Survey (ATUS)		United Kingdom Time Use Survey (UKTUS)		French Time Use Survey	
	Mean	(s.e.)	Mean	(s.e.)	Mean	(s.e.)	Mean	(s.e.)
How time is spent: difference in time spent per week (hours)								
Home production	− 5.16	(0.71)	− 5.78	(0.67)	− 2.22	(1.45)	− 4.66	(1.06)
Sleep	0.92	(0.49)	1.30	(0.50)	− 2.13	(1.07)	0.38	(0.95)
Other personal	− 0.31	(0.33)	− 0.18	(0.40)	− 0.71	(0.90)	− 3.16	(0.75)
TV watching	0.59	(0.56)	3.05	(0.73)	− 1.00	(1.56)	1.55	(1.02)
Other leisure	3.96	(0.66)	1.60	(0.77)	6.04	(1.52)	5.89	(1.06)
Who time is spent with: difference in proportion of time spent (%)								
With spouse	− 53.04	(5.05)	− 50.67	(5.55)	− 68.52	(23.15)	− 55.09	(7.02)
Alone	37.93	(5.76)	36.95	(6.58)	57.67	(29.81)	38.18	(10.00)
With others	15.11	(3.80)	13.72	(4.07)	10.85	(28.25)	16.91	(6.38)
Number of observations (diaries)	5291		4124		634		2174	
Number of individuals	2668		4124		634		1093	

Samples include all women aged 70+ for whom necessary control variables were available. Differences controlling for year and month of interview, age, education, and income. For Polish Time Use Survey 2013 controlling also for day of the week, region, size of city, immigrant status, and income squared, and, if available, diaries from two different days included per person. For ATUS data controlling also for census region, rural location, immigrant status, race/ethnicity and health status, and computed on observations from years 2006–2008, 2010–2014, 2016 for which all control information is available. For French Time Use Survey 2009–10 controlling also for region, size of city, income squared, general health, type of home ownership, and, if available, diaries from two different days included per person. For UKTUS data computed for years 2014–2015

**Table 5:** Time use survey sub-samples: descriptive statistics. *Source*: own calculations based on Polish (2013), American (2012–2013), United Kingdom (2014–2015), French (2009–10) Time Use Survey data

	Polish Time Use Survey		American Time Use Survey (ATUS)		United Kingdom Time Use Survey (UKTUS)		French Time Use Survey	
Number of individuals	2668		888		276		103	
Marital status (%)								
Widowed	1897	(71.1%)	462	(52.0%)	129	(46.6%)	52	(50.5%)
Other	771	(28.9%)	426	(48.0%)	147	(53.4%)	51	(49.5%)
Life satisfaction (%)								
Satisfied	1889	(70.8%)	599	(67.4%)	192	(69.7%)	73	(71.1%)
Not satisfied	779	(29.2%)	289	(32.6%)	84	(30.3%)	30	(28.9%)
Mean age (SD)	77.10	(5.30)	77.26	(4.54)	77.34	(5.18)	78.64	(5.77)
Mean family income per year (SD)	19,092 PLN	(8570 PLN)	$41,075	($36,430)	₤28,978	(₤75,087)	₤16,236	(₤6865)
Number of diaries	5291		888		276		206	
Mean time (hours/day) spent on (SD)								
Home production	4.47	(2.39)	4.04	(3.01)	5.30	(2.36)	4.33	(2.34)
Sleep	9.60	(1.57)	9.06	(2.09)	8.74	(1.80)	8.93	(1.80)
Other personal	2.98	(1.09)	2.23	(1.52)	3.27	(1.33)	3.73	(1.66)
TV watching	3.14	(1.86)	4.22	(3.16)	3.14	(2.36)	2.68	(1.83)
Other leisure	3.82	(2.26)	4.45	(3.29)	3.55	(2.36)	4.33	(2.01)
Mean time (hours/day) spent (SD)								
Alone	9.73	(4.05)	7.93	(4.81)	10.18	(8.46)	7.99	(4.84)
With others (non-spouse)	2.40	(2.74)	1.98	(3.00)	3.73	(6.99)	1.94	(2.89)
With spouse (married only)	7.84	(3.58)	6.42	(4.23)	15.28	(6.64)	8.42	(3.52)

‘Satisfied with life’ = 1 if > 3 on a 5–1 scale in the Polish Time Use Survey, > 7 on a 10–0 life satisfaction scale in the ATUS, > 5 on a 7–1 scale in the UKTUS, and > 6 on a 10–0 scale in the French Time Use Survey. Life satisfaction in ATUS was collected only in years 2012–2013; in UKTUS and French Time Use Survey it was collected only for a subsample of participants

### Outcomes analyzed in the study

The primary outcome is the association between widowhood and well-being. Life satisfaction is a common measure of subjective well-being and it has been reported in all surveys used in the analysis. Different surveys, however, apply different scales to measure life satisfaction. In the SHARE survey and in the American and French Time Use Surveys an 11-point Cantril Ladder was used, an 8-point scale was implemented in the U.K. Time Use Survey, and a 5-point scale was applied in the Polish Time Use Survey.^6^

For the regression analysis we construct a binary indicator of life satisfaction that designates about 2/3 of a specific sample as satisfied with life. Survey participants who are identified as satisfied with life scored 8 points or more on the 11-point scale in the SHARE survey and the American Time Use Survey, 7 points or more on the 11-point scale in the French Time Use Survey, 6 points or more on the 8-point scale in the U.K. Time Use Survey, and 4 points and more on the 5-point scale in the Polish Time Use Survey.^7^

Apart from life satisfaction, SHARE collects several other indicators of well-being. We take advantage of measures of mental health which in SHARE are captured through the EURO-D scale of depression, an international scale developed specifically to evaluate symptoms of depression among older European populations (Guerra et al., 2015; Prince et al., 1999). The scale is composed of 12 items. We use several items from this scale and a binary indicator of the risk of depression, based on a threshold of four or more symptoms, as commonly utilized in the literature (Choi et al., 2013; Guerra et al., 2015; Richardson et al., 2020). Measures of life satisfaction and measures of depression are separate indicators of well-being—they need not be positively correlated, nor do the impacts of demographic and other indicators necessarily affect them in the same directions. However, if we were to observe substantially different effects on these two outcomes, one could be concerned about how general our results are. By examining how both types of measures change over time, using the same data and methods, our estimates allow us to obtain a more general indication of differences in well-being between widows and other older women.

## Results

**Fig. 2:**
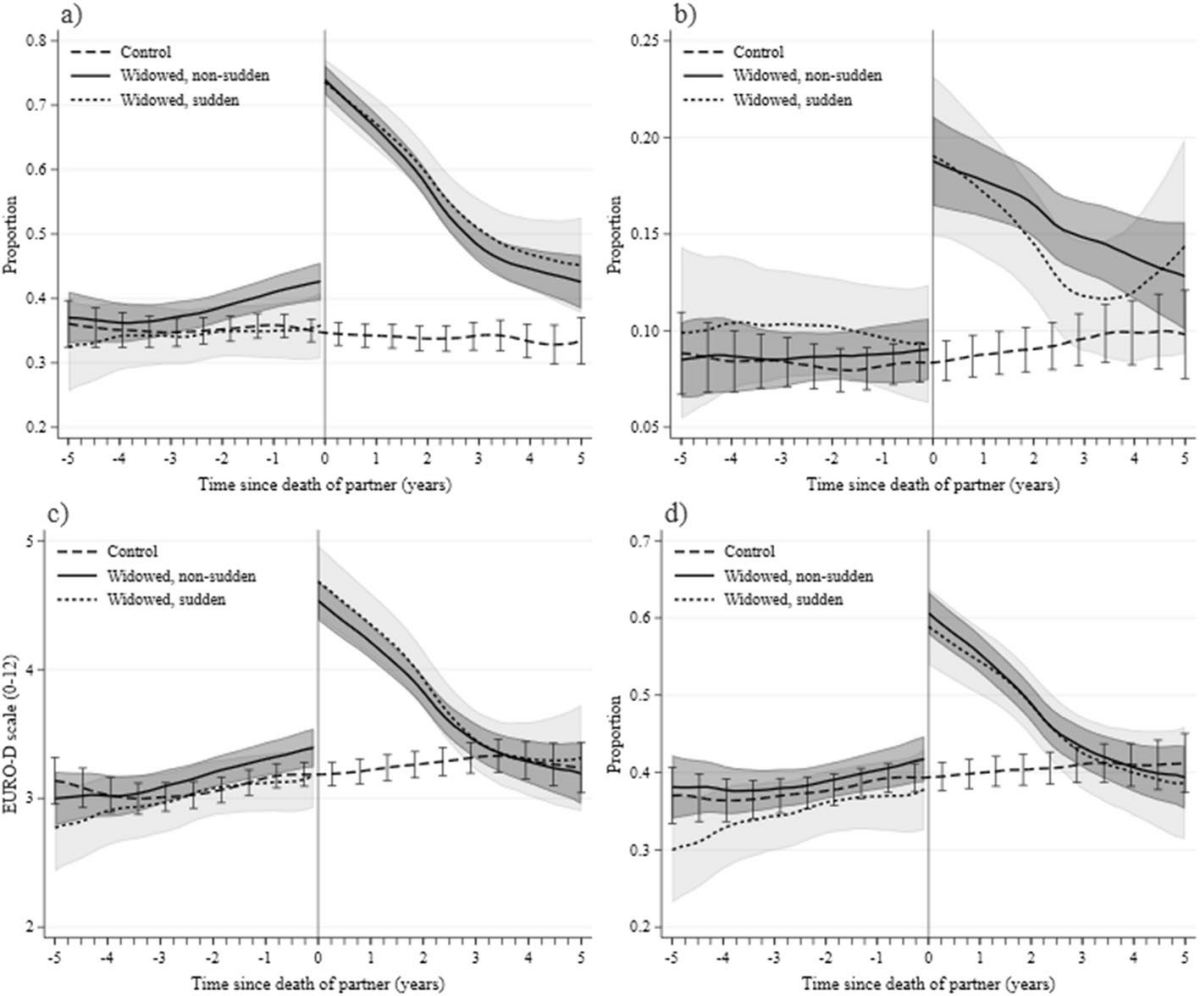
Depressive symptoms before and after partner’s death. In the last month: **a** “cried at all”; **b** “felt that would rather be dead”. EURO-D depression scale: **c** Number of symptoms (0–12); **d** Depression risk (4+ symptoms). *Source*: own calculations based on SHARE data. *Note*: Number of individuals for control/non-sudden/sudden samples = a: 2288/1725/563; b: 2284/1722/562, c, d: 2258/1704/554. Each individual is observed at least twice—before and after partner’s death (actual or imputed for controls)

We first analyze the development of widows’ well-being before and after their partner’s death with reference to several measures of mental health based on the EURO-D depression scale. Figure 2 presents their evolution: frequency of crying (Fig. 2a); suicidal thoughts (Fig. 2b); the total reported number of symptoms of depression (Fig. 2c); and the likelihood of reporting four or more items, used to measure the risk of depression (Fig. 2d). In each case the path is shown before and after time 0—the date of the partner’s death and the matched date for controls—for three groups: non-sudden widows (black solid line), sudden widows (black short-dashed line), and controls (black dashed line).^8^

Figure 2a shows the proportion of females crying in the last month. Among non-widowed controls, around 35% reported crying, with little variation over time. Among non-sudden widows this percentage is already significantly higher than among controls 2 years before widowhood, while among sudden widows it is not significantly different from the control group before widowhood. This proportion increases to over 70% crying after the partner’s death for all widows. Moreover, the implications of losing one’s partner are long-lasting: 2 years after the death, around 60% of widows still reported crying in the previous month. Becoming widowed affects also other aspects of mental health, including feeling that one would “rather be dead” (Fig. 2b). The number of symptoms of depression on the 12-symptom EURO-D scale (Fig. 2c) and the likelihood of suffering four or more symptoms of depression (Fig. 2d) also increase upon widowhood in a similar manner.

**Fig. 3:**
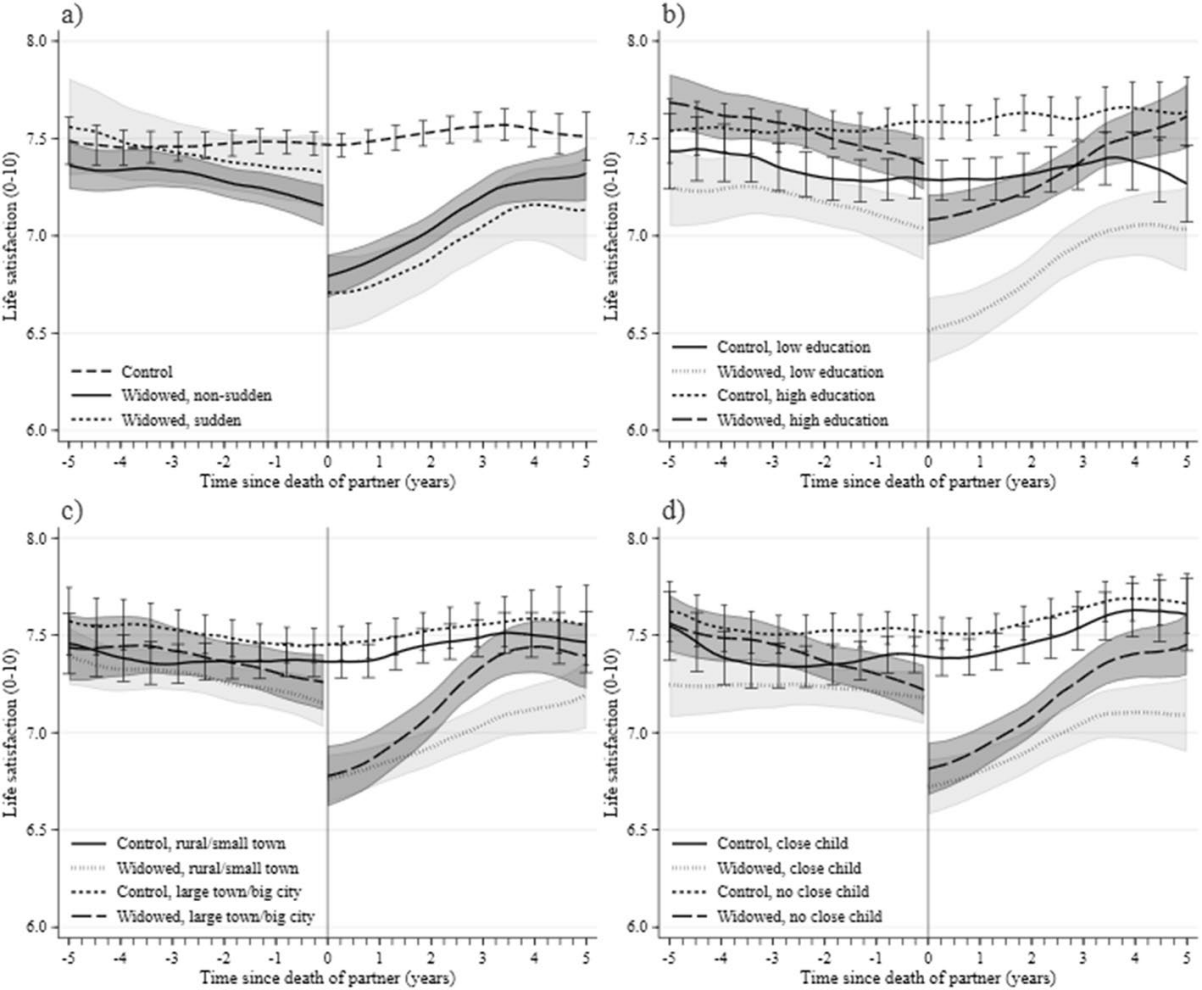
Life satisfaction before and after partner’s death. **a** Widows due to sudden and non-sudden death. **b** Widows by education status. **c** Widows by residence status. **d** Widows with and without children living close. *Source*: own calculations based on SHARE data. *Note*: Life satisfaction on a 10–0 scale. **b** Low education: no education, primary education or still in school; high education: upper secondary education or higher; lower secondary education left out. **c** Rural/small town: rural areas and small towns. **d** Close child: one or more in same household/building or less than 1 km away, contacts at least once weekly. Number of individuals in the samples sequenced as in the legends = a: 2814/2142/672; b: 1042/1065/1227/1188; c: 1530/1590/1275/1215; d: 1290/1333/1506/1463. Each individual is observed at least twice—before and after partner’s death (actual or imputed for controls)

Figure 3 presents the evolution of life satisfaction rated in the SHARE survey on the 10 to 0 scale, again with the time of the partner’s death denoted as time 0. In Fig. 3a, as in Fig. 2, we differentiate between sudden and non-sudden widows. The dynamics of life satisfaction among the widowed sample before sudden widowhood closely matches that of the controls. Among non-sudden widows the match is initially very close, but, as in the case of tearfulness (Fig. 2a), the level of life satisfaction begins to diverge from that of controls much earlier—already around 3 years before the death. The most likely causes of this relative decline are concerns about a partner’s deteriorating condition and the burden of caring obligations. Following the partner’s death, widows unsurprisingly exhibit much reduced satisfaction with their lives. In both groups they evaluate their lives more favorably as time passes; but even after four-and-a-half years of widowhood their life satisfaction remains below that of controls. As late as 3 years following the death of their partner, non-sudden widows have recovered only 50% and sudden widows only 36% of the gap in life satisfaction.

The results on life satisfaction parallel our estimates for measures of depression. Similar patterns of differences between widowed and married women are observed before and after a woman is widowed. Moreover, unpublished regression estimates demonstrate that the same covariates that significantly alter depression affect life satisfaction in the same directions.

We next study the drop in life satisfaction among various subgroups to identify potential factors moderating the implications of widowhood. Figure 3b shows that declines are larger among less-educated than more-educated widows, and that the magnitude of the decline is greater than the difference in life satisfaction between these groups before the partner’s death. This comparison shows how dramatic the decline in well-being after a partner’s loss is—given how strongly education differentiates people with respect to income, wealth, and health. Figure 3c demonstrates the differences in experiencing widowhood between urban and rural areas and shows that the life satisfaction of widows residing in urban areas recovers more rapidly.

In Fig. 3d the sample is divided based on the physical proximity and frequency of the woman’s contact with children, which could be expected to be a decisive factor determining the evolution of widows’ well-being. In this case we note two major results. On the one hand, the differences in life satisfaction before time 0 between future widows and controls are only statistically significant among those without children living close by. This may reflect the support which mothers receive from their nearby children in the last years before the death of the partner, thus reducing the negative impact on the mother’s well-being. On the other hand, the drop in life satisfaction among widows who lived close to at least one of their children is greater than that of other widows; and, in addition, it recovers more slowly. This suggests that the existence and physical proximity of family members is not by itself sufficient to ameliorate the drop in well-being in widowhood.^9^

As pointed out in the literature (Jeon et al., 2013; Li et al., 2005; Subramanian et al., 2008; Utz et al., 2014), another factor that might differentiate widows’ well-being is the social network. The SHARE dataset collects information about the family relations of its respondents which, beyond simple enumeration of household or family members, also includes the strength of the relationship. Using the indicator of the number of people with whom participants state that they “often discuss things that are important,” we estimate regressions also controlling for such features as having at least one person in the social network, having the partner, a child, or a friend with whom one feels close listed in it, and for satisfaction with the network.

**Fig. 4:**
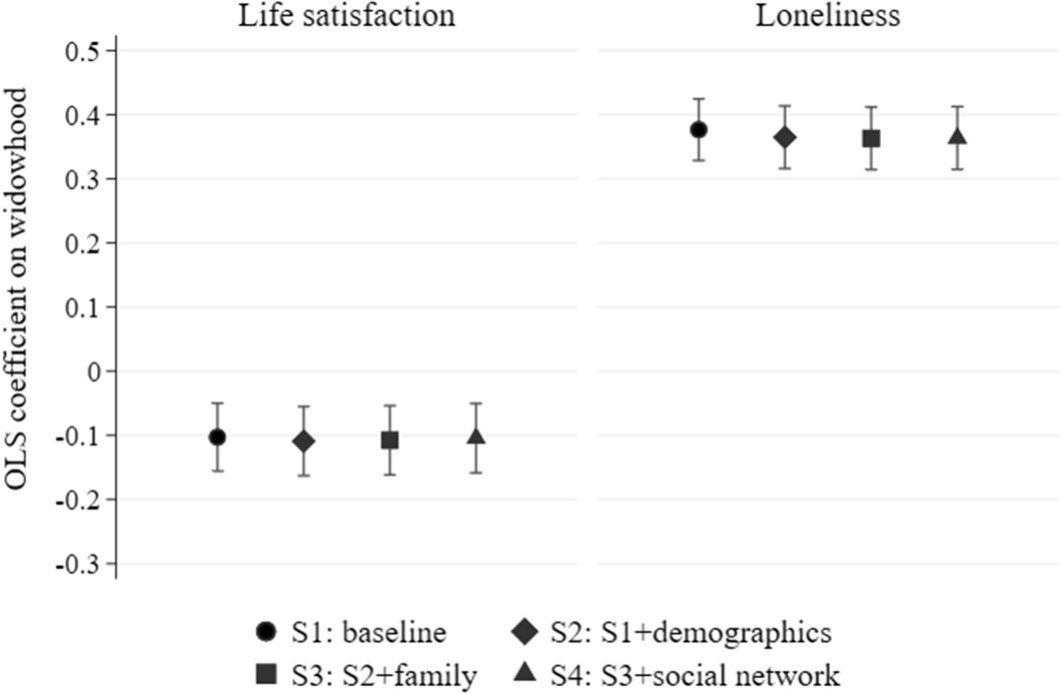
Widowhood, life satisfaction, and loneliness: family and the social network in the SHARE survey. *Source*: own calculations based on SHARE data. *Note*: OLS regressions on widowhood indicator (coefficients with 95% confidence intervals). Life satisfaction = 1 if ≥ 8 on a 10–0 scale. Loneliness = 1 if felt lonely often or some of the time. Specifications 1–4 include country controls, Specifications 2–4 add interview month, age, education, health, residence, home ownership; Specifications 3–4 add number of children, contact/distance to children; Specification 4 adds social network. Complete results of the regressions in all specifications are available in Tables S5–S6 in the online Supplementary Information. Number of individuals = 3056

Figure 4 presents widow-control differences in life satisfaction and feelings of loneliness in four specifications: the raw difference (specification S1); regressions adjusted for basic demographics (specification S2); adjusted for the effect of respondents’ nearby children (specification S3), and finally also adjusted for women’s pre-widowhood social networks (specification S4). Confirming the findings of Fig. 3d, differences in family circumstances or in the character of the social network cannot explain the effects of widowhood on life satisfaction—the estimated effects change only slightly between specifications S1 and S4. The second panel of Fig. 4 shows further that feelings of loneliness in widowhood are also nearly independent of family circumstances and the broader pre-widowhood social network. The existence of these social relations and the differences in the size and nature of the social network thus have no explanatory power regarding the drop in life satisfaction upon widowhood. We should stress that these are feelings of being alone—subjective measures, and they say nothing about whether these widows are with other people less than married women.

To identify the conditions which are responsible for this drop we examine data showing how widows use their time compared to partnered women, taking advantage of the time-diaries. As shown in Table 4, widows spend their time differently from otherwise identical partnered older women: they spend less time in home production, and more time on ‘other leisure’. They can no longer spend time with their partners, which is the predominant category for those who continue to live in couples: over 50% in France, Poland, and the U.S. and almost 70% in the U.K. In turn, widows spend much more time alone—an additional 40–60% in these countries compared to partnered women—and a little more time with other people.

**Fig. 5:**
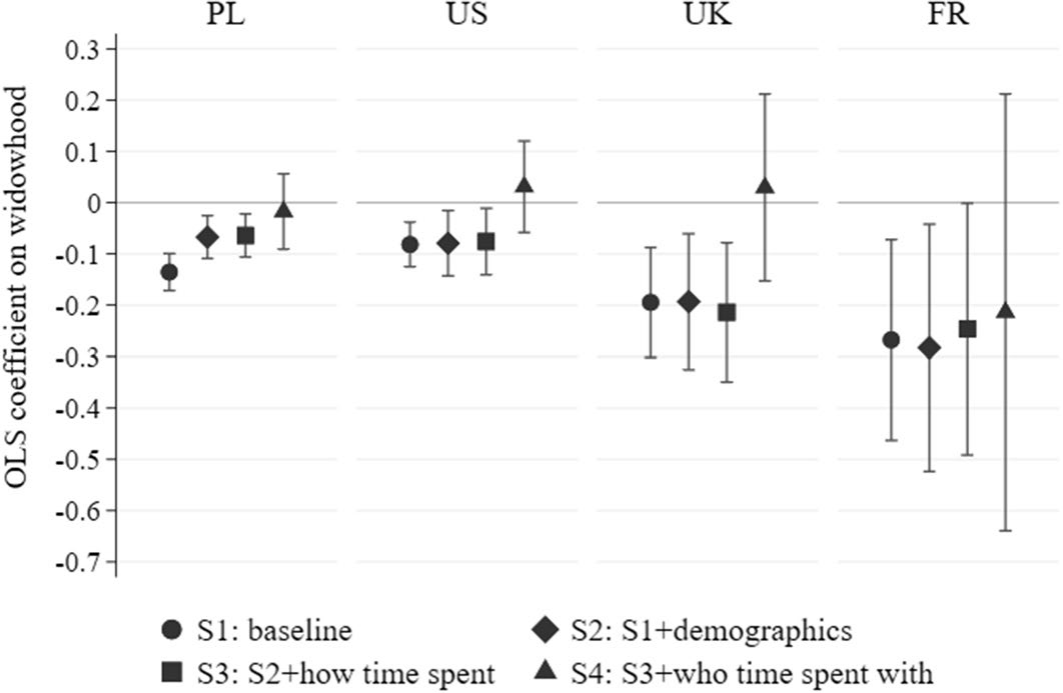
Time use and life satisfaction in Poland, the U.S., the U.K. and France. *Source*: own calculations based on Polish (2013), American (2012–2013), United Kingdom (2014–2015), French (2009–10) Time Use Survey data. *Note*: OLS regressions on widowhood indicator (coefficients with 95% confidence intervals). Life satisfaction = 1 if ≥ 4 on a 5–1 scale (PL), ≥ 8 on a 10–0 scale (U.S), ≥ 6 on a 7–1 scale (U.K.), and ≥ 7 on a 10–0 scale (FR). Specifications 2–4 add date and weekday of interview, age, education, income, and some additional controls depending on country; Specifications 3–4 add time spent in each activity; Specification 4 adds who with the time was spent. Complete results of the regressions in all specifications are available in Tables S7–S10 in the Supplementary Information. Number of observations in the PL/U.S./U.K./FR sample = 5291/888/276/206. Countries ordered according to the size of the sample and respectively CIs

The time-use datasets employed here further allow for examining of how time use affects life satisfaction. The results are shown in Fig. 5. We compare life satisfaction between partnered and widowed women, successively expanding the set of control variables, as in Fig. 4. For each country, the left-most point (specification S1) in Fig. 5 shows the raw difference. The results from Poland and the U.S. closely mirror those in Figs. 3 and 4. Differences in the U.K. and France are larger, although estimated less precisely, as the available samples from these two countries are smaller. Regressions adjusted for a large set of socio-demographic differences—potentially moderating the effect of widowhood (specification S2)—hardly reduce the estimated shortfall in widows’ life satisfaction. By further extending the set of control variables we account for different conditions in widowhood from the point of view of time use, thus examining factors which might mediate the implications of losing a partner. In particular, in specification S3 we show that the differences in life satisfaction do not arise because widows spend time differently, and what matters is who the widow spends time with, as shown in specification S4. In these regressions, the differences in life satisfaction between widows and otherwise identical married older women are driven down to zero, with the exception of France, where the small sample size prevents precise estimation. By including a measure of time actually spent alone, these findings complement those in Fig. 4. They imply that feelings of loneliness result from the widows actually being alone—spending more of their time by themselves—compared to married women. Feelings of loneliness and actually being alone are not the same thing; but our results show that widowhood correlates strongly with both, and that being alone is a key driver of widows’ lower life satisfaction.

While only in Poland does the sample size allow us to differentiate statistically between specifications 1 and 4, the pattern of reactions of the point estimates in all four countries seems very clear. It is also very different when compared to the stability of its values presented in Fig. 4 and to the estimated values in specifications 2 and 3 of Fig. 5. Thus, although insufficient statistical power suggests treating our conclusions with some caution, of the extensive set of covariates that we examine, only the measure of time spent explains widows’ reduced levels of well-being.

## Conclusion

We show that widows’ well-being, as reflected in their life satisfaction and indicators of their mental health, drops significantly upon their partner’s passing and recovers only slowly. This drop affects widows across different social groups and cannot be fully explained by variations in family circumstances. Along several dimensions, widows’ well-being remains lower than controls’ at least 5 years after the partner’s death, and the recovery path is slower among those who lost their partners suddenly, which confirms slower adaptation to widowhood. Three years after the death of their partner, suddenly-widowed women have recovered only about a third of the gap in life satisfaction relative to the control sample. We demonstrate that the implications of widowhood cannot be explained by differences in family structure, proximity of children, or the size and nature of widows’ social networks.

Time-use data from several European countries and the U.S. show that the key factor is that widows are alone substantially more than married older women. Socio-demographic characteristics, family circumstances, their social network before being widowed, and the types of their daily activities do not reduce the shortfall in life satisfaction. Their greater time spent alone is the sole identifiable cause. It is likely to be the key factor behind increased feelings of loneliness in widowhood and an important contributor to worsened mental health.

These results should be interpreted with some caution. Long panel studies such as SHARE survey commonly suffer from non-random cumulative attrition, a problem which cannot be easily accounted for in the analysis, though a lot of effort is devoted to decrease its scope already at the stage of fieldwork (i., e., proxy interviews with help of close family members, a special protocol to follow individuals who moved to nursing homes). It is worth noting though, that if attrition is in any way specifically related to widowhood—for example, through the potential effect of partner’s death on widows’ health—then the effects of widowhood identified in our analysis would reflect a lower bound of its negative consequences.

Our findings suggest that the key aspect to understanding lower well-being in widowhood is being alone and that reduced well-being among surviving partners persists far beyond the initial months of widowhood. It is difficult to imagine a time more sensitive than during the mourning for a partner. Through carefully constructed policies that reduce time spent alone and facilitate greater active social interactions, widows’ well-being could be increased, and the time during widowhood when they are dissatisfied with their lives could be shortened. Even though proximity of children—not the amount of time spent with them—seems not to affect widows’ well-being, the time-diary evidence suggests actually spending time with them (and others) does. The seemingly inevitable drop in well-being among widows generally and especially those who lost their partners to the COVID-19 pandemic could be vitiated if women were not left to face widowhood alone.

## Supplementary Information

Below is the link to the electronic supplementary material.

Supplementary file1 (DOCX 254 kb)

## Data Availability

Data underlying the analysis conducted in this paper is available for scientific purposes at www.share-project.org (SHARE data), ukdataservice.ac.uk (UKTUS data), www.atusdata.org/atus/ (ATUS data), and on request from the French Data Archives for Social Sciences ADISP (French Time Use data) and from the Polish Central Statistical Office (Polish Time Use data). The analysis presented in this manuscript was prepared using Stata 15 software. Files with syntax enabling replication are available under the following link: https://dx.doi.org/10.17632/9xfgfrywcm.1

## Footnotes

1 Table S2 in the online Supplementary Information gives an overview of the total number of different types of interviews collected in each participating country across all seven waves of the study conducted between 2004 and 2017.

2 While SHARE is a biennial survey, it might happen that the time between two consecutive waves is shorter than 2 years in some cases. This might be due to extensions of fieldwork in some years, different starting times or interviews with specific households taking place at different stages of survey waves.

3 Table S3 in the online Supplementary Information gives an overview of the balance between the widowed and control samples. The distribution of the propensity score of the matched samples of widowed and non-widowed individuals is presented in Figure S2.

4 We tested several other matching approaches with additional characteristics for the exact stage with very little difference in the results. We therefore limit the exact stage only to country matching to minimize the loss of observations due to lack of common support. Similarly, we tested different calipers to avoid the problem of increasing imbalance in the sample (King & Nielsen, 2019).

5 To our knowledge these are the only surveys with a question on life satisfaction asked alongside the details on time use and other contextual information including income, which is crucial for the analysis.

6 Table S4 in the online Supplementary Information provides details on the exact construction of questions and answer options in all of these surveys.

7 Our main conclusions are robust to using reasonable different thresholds, including cut-offs of 6 or 7 on the SHARE and the ATUS.

8 Results based on the SHARE data show overall effects for the entire international sample, and thus reflect ‘average’ levels and patterns of development for the examined outcomes. Detailed country-specific analyses—where possible, given the sizes of individual country samples—show similar evolutions of the outcomes in most of the countries. In several cases (e.g., Greece and Denmark) relative reactions to widowhood vary across outcomes, and only in Switzerland do we find almost no reduction of life satisfaction with widowhood.

9 Robustness checks which split the sample by the gender of the child who lived closest show little difference in the evolution of well-being of between mothers of sons and mothers of daughters.
