# Supplementary material for: Home Alone: Widows’ Well-Being and Time
Source: J Happiness Stud. 2023 Jan 20;24(2):813–38. doi: 10.1007/s10902-023-00622-w (PMC9851896; doi:10.1007/s10902-023-00622-w)
Supplement: Supplementary file 1 — Supplementary file1 (DOCX 254 kb) [file 10902_2023_622_MOESM1_ESM.docx]

**Home alone: Widows' well-being and time**

**Supplementary Information**

**Figure S1 Change in the number of deceased married men aged 70+ in selected countries in the period of 2008 to 2019 as compared to 2007**

*
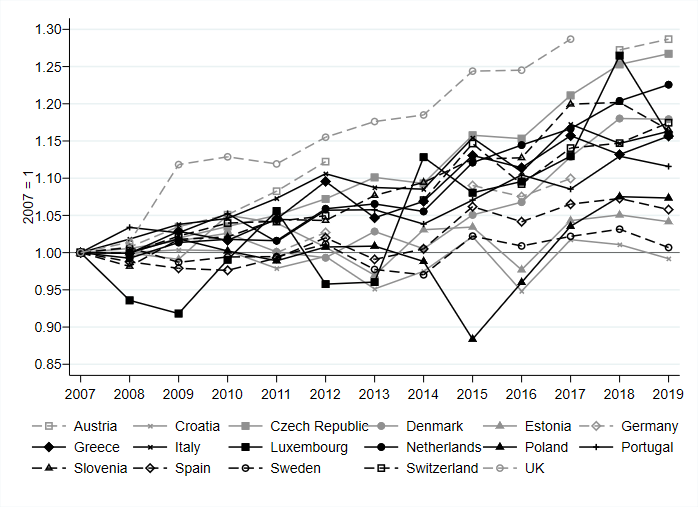
*

Source: EUROSTAT.

Notes: Countries used for analysis in the manuscript, except for Belgium, France, Israel (no data). Single years missing in case of some countries (Austria, Germany, Poland, Switzerland). Due to missing 2007 information, for Germany 2011 used as the reference year, for Poland – 2008.

**Figure S2 Matching widowed and non-widowed individuals: distribution of the propensity score (SHARE)**


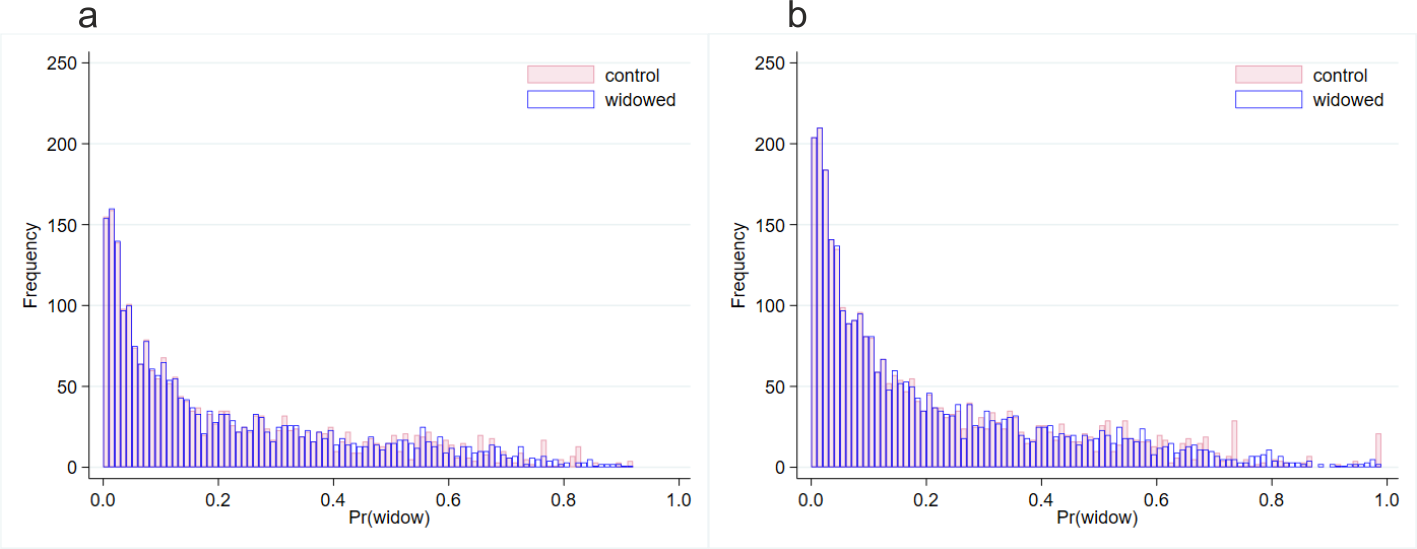


Source: own calculations based on SHARE data.

Note: (a) Sample for the analysis of mental health outcomes: 2381 control/widowed observations. (b) Sample for the analysis of life satisfaction: 3076 control/widowed observations.

**Table S1 Deceased married men aged 70+ in selected countries in 2007 and 2019 (total number and share of adult male population)**

|  | 2007 | | 2019 | |
| --- | --- | --- | --- | --- |
| Country | Total number | % of adult male  population | Total number | % of adult male  population |
| Austria | 13535 | 0.40% | 17417 | 0.47% |
| Croatia | 9522 | 0.55% | 9445 | 0.57% |
| Czech Republic | 17036 | 0.40% | 21588 | 0.49% |
| Denmark | 8702 | 0.40% | 10262 | 0.43% |
| Estonia | 2211 | 0.42% | 2303 | 0.45% |
| Germany* | 177938 | 0.53% | 195657 | 0.56% |
| Greece | 26788 | 0.58% | 30981 | 0.70% |
| Italy | 132264 | 0.55% | 153822 | 0.61% |
| Luxembourg | 733 | 0.38% | 849 | 0.33% |
| Netherlands | 25731 | 0.39% | 31537 | 0.44% |
| Poland* | 62174 | 0.40% | 66736 | 0.43% |
| Portugal | 21907 | 0.52% | 24444 | 0.59% |
| Slovenia | 3359 | 0.40% | 3913 | 0.45% |
| Spain | 87245 | 0.46% | 92293 | 0.47% |
| Sweden | 15933 | 0.43% | 16042 | 0.38% |
| Switzerland | 12193 | 0.40% | 14321 | 0.40% |
| UK* | 88313 | 0.36% | 113645 | 0.43% |

Source: EUROSTAT.

Notes: Countries used for analysis in the manuscript, except for Belgium, France, Israel (no data on deceased married men). Adult male population defined as men aged 15 years or more. * - due to missing information in the number of deceased married men, in Germany 2011 data reported instead of 2007 and 2017 instead of 2019, in Poland 2008 instead of 2007, in the UK 2017 instead of 2019.

**Table S2 Overall number of interviews in waves 1-7 of SHARE across countries**

|  | **Wave1** | **Wave 2** | | **Wave 3** | | **Wave 4** | | **Wave 5** | | **Wave 6** | | **Wave 7** | | |
| --- | --- | --- | --- | --- | --- | --- | --- | --- | --- | --- | --- | --- | --- | --- |
| **Country** | Main | Main | EOL | SHARE-Life | EOL | Main | EOL | Main | EOL | Main | EOL | Main | SHARE-Life | EOL |
|  |  |  |  |  |  |  |  |  |  |  |  |  |  |  |
| Austria | 1563 | 1197 | 36 | 999 | 50 | 5247 | 45 | 4378 | 178 | 3397 | 158 | 483 | 2723 | 179 |
| Germany | 2995 | 2628 | 52 | 1919 | 68 | 1619 | 54 | 5751 | 31 | 4412 | 99 | 836 | 2985 | 126 |
| Sweden | 3049 | 2796 | 63 | 1961 | 101 | 1969 | 182 | 4556 | 189 | 3906 | 176 | 1066 | 2131 | 151 |
| Netherlands | 2968 | 2683 | 49 | 2258 | 83 | 2789 | 61 | 4168 | 85 | 0 | 0 | 0 | 0 | 0 |
| Spain | 2316 | 2423 | 97 | 2271 | 138 | 3727 | 142 | 6693 | 268 | 5623 | 425 | 1280 | 3431 | 432 |
| Italy | 2552 | 2984 | 52 | 2528 | 92 | 3570 | 92 | 4745 | 152 | 5311 | 240 | 1570 | 3001 | 237 |
| France | 3122 | 2990 | 59 | 2500 | 112 | 5850 | 101 | 4506 | 144 | 3947 | 123 | 1143 | 2188 | 191 |
| Denmark | 1706 | 2630 | 50 | 2144 | 91 | 2287 | 121 | 4146 | 133 | 3733 | 181 | 1284 | 1962 | 164 |
| Greece | 2897 | 3412 | 50 | 3092 | 145 | 0 | 0 | 0 | 0 | 4928 | 350 | 1911 | 1161 | 272 |
| Switzerland | 997 | 1498 | 14 | 1324 | 26 | 3786 | 39 | 3049 | 65 | 2803 | 93 | 754 | 1648 | 86 |
| Belgium | 3810 | 3227 | 40 | 2865 | 104 | 5322 | 91 | 5637 | 152 | 5815 | 227 | 1567 | 3335 | 205 |
| Israel | 2449 | 2447 | 164 | 0 | 0 | 0 | 0 | 2599 | 195 | 2035 | 155 | 0 | 2132 | 124 |
| Czech Rep. | 0 | 2736 | 0 | 1817 | 67 | 5526 | 81 | 5640 | 223 | 4856 | 324 | 921 | 3298 | 293 |
| Poland | 0 | 2466 | 0 | 1939 | 94 | 1733 | 165 | 0 | 0 | 1826 | 195 | 1144 | 3559 | 113 |
| Ireland | 0 | 1035 | 0 | 855 | 36 | 0 | 0 | 0 | 0 | 0 | 0 | 0 | 0 | 0 |
| Luxembourg | 0 | 0 | 0 | 0 | 0 | 0 | 0 | 1610 | 0 | 1564 | 18 | 0 | 1254 | 35 |
| Hungary | 0 | 0 | 0 | 0 | 0 | 3072 | 0 | 0 | 0 | 0 | 0 | 0 | 1538 | 300 |
| Portugal | 0 | 0 | 0 | 0 | 0 | 2013 | 0 | 0 | 0 | 1674 | 116 | 0 | 1282 | 88 |
| Slovenia | 0 | 0 | 0 | 0 | 0 | 2748 | 0 | 2958 | 52 | 4223 | 122 | 0 | 3691 | 191 |
| Estonia | 0 | 0 | 0 | 0 | 0 | 6863 | 0 | 5752 | 331 | 5638 | 368 | 0 | 5116 | 376 |
| Croatia | 0 | 0 | 0 | 0 | 0 | 0 | 0 | 0 | 0 | 2495 | 0 | 0 | 2408 | 101 |
| Lithuania | 0 | 0 | 0 | 0 | 0 | 0 | 0 | 0 | 0 | 0 | 0 | 0 | 2035 | 0 |
| Bulgaria | 0 | 0 | 0 | 0 | 0 | 0 | 0 | 0 | 0 | 0 | 0 | 0 | 1998 | 0 |
| Cyprus | 0 | 0 | 0 | 0 | 0 | 0 | 0 | 0 | 0 | 0 | 0 | 0 | 1233 | 0 |
| Finland | 0 | 0 | 0 | 0 | 0 | 0 | 0 | 0 | 0 | 0 | 0 | 0 | 2007 | 0 |
| Latvia | 0 | 0 | 0 | 0 | 0 | 0 | 0 | 0 | 0 | 0 | 0 | 0 | 1734 | 0 |
| Malta | 0 | 0 | 0 | 0 | 0 | 0 | 0 | 0 | 0 | 0 | 0 | 0 | 1261 | 0 |
| Romania | 0 | 0 | 0 | 0 | 0 | 0 | 0 | 0 | 0 | 0 | 0 | 0 | 2114 | 0 |
| Slovakia | 0 | 0 | 0 | 0 | 0 | 0 | 0 | 0 | 0 | 0 | 0 | 0 | 2077 | 0 |
|  |  |  |  |  |  |  |  |  |  |  |  |  |  |  |
| Total | 30424 | 37152 | 726 | 28472 | 1207 | 58121 | 1174 | 66188 | 2198 | 68186 | 3370 | 13959 | 63302 | 3664 |

Source: own compilation based on SHARE data.

Note: Main – regular interview; EOL – *end-of-life* interview conducted for a deceased participant from previous wave(s); SHARE-Life – an interview capturing participant’s life history. Participants in wave 7 are split between regular interviews (main) and SHARE-Life interviews. Countries ordered based on their first participation and SHARE ordering scheme.

**Table S3 Balancing table for final matched samples for the analysis of mental health outcomes and the analysis of life satisfaction (SHARE)**

|  | **Sample for Figures 2A-D** | | | | **Sample for Figures 3A-D** | | | |
| --- | --- | --- | --- | --- | --- | --- | --- | --- |
|  | **Mean value control** | **Mean value widowed** | **t** | **p** | **Mean value control** | **Mean value widowed** | **t** | **p** |
| Time btw. before/after interview | 41.03 | 41.49 | -0.66 | 0.51 | 38.48 | 39.23 | -1.28 | 0.20 |
| Age (initial wave) | 69.84 | 70.19 | -1.41 | 0.16 | 70.31 | 70.56 | -1.15 | 0.25 |
| Education: |  |  |  |  |  |  |  |  |
| - Primary or less | 0.41 | 0.41 | -0.06 | 0.95 | 0.39 | 0.40 | -0.52 | 0.60 |
| - Secondary and post-secondary non-tertiary | 0.46 | 0.46 | -0.20 | 0.84 | 0.47 | 0.47 | -0.49 | 0.63 |
| - Tertiary | 0.11 | 0.11 | -0.05 | 0.96 | 0.12 | 0.12 | 0.94 | 0.35 |
| - Other | 0.02 | 0.02 | 1.22 | 0.22 | 0.02 | 0.01 | 1.66 | 0.10 |
| Country: |  |  |  |  |  |  |  |  |
| - Austria | 0.04 | 0.04 | 0.00 | 1.00 | 0.05 | 0.05 | 0.00 | 1.00 |
| - Germany | 0.05 | 0.05 | 0.00 | 1.00 | 0.05 | 0.05 | 0.00 | 1.00 |
| - Sweden | 0.07 | 0.07 | 0.00 | 1.00 | 0.06 | 0.06 | 0.00 | 1.00 |
| - Netherlands | 0.04 | 0.04 | 0.00 | 1.00 | 0.03 | 0.03 | 0.00 | 1.00 |
| - Spain | 0.11 | 0.11 | 0.00 | 1.00 | 0.12 | 0.12 | 0.00 | 1.00 |
| - Italy | 0.10 | 0.10 | 0.00 | 1.00 | 0.08 | 0.08 | 0.00 | 1.00 |
| - France | 0.06 | 0.06 | 0.00 | 1.00 | 0.06 | 0.06 | 0.00 | 1.00 |
| - Denmark | 0.06 | 0.06 | 0.00 | 1.00 | 0.05 | 0.05 | 0.00 | 1.00 |
| - Greece | 0.06 | 0.06 | 0.00 | 1.00 | 0.06 | 0.06 | 0.00 | 1.00 |
| - Switzerland | 0.03 | 0.03 | 0.00 | 1.00 | 0.03 | 0.03 | 0.00 | 1.00 |
| - Belgium | 0.08 | 0.08 | 0.00 | 1.00 | 0.06 | 0.06 | 0.00 | 1.00 |
| - Israel | 0.05 | 0.05 | 0.00 | 1.00 | 0.05 | 0.05 | 0.00 | 1.00 |
| - Czech Republic | 0.08 | 0.08 | 0.00 | 1.00 | 0.08 | 0.08 | 0.00 | 1.00 |
| - Poland | 0.06 | 0.06 | 0.00 | 1.00 | 0.05 | 0.05 | 0.00 | 1.00 |
| - Luxembourg | 0.00 | 0.00 | 0.00 | 1.00 | 0.01 | 0.01 | 0.00 | 1.00 |
| - Portugal | 0.01 | 0.01 | 0.00 | 1.00 | 0.02 | 0.02 | 0.00 | 1.00 |
| - Slovenia | 0.02 | 0.02 | 0.00 | 1.00 | 0.03 | 0.03 | 0.00 | 1.00 |
| - Estonia | 0.08 | 0.08 | 0.00 | 1.00 | 0.10 | 0.10 | 0.00 | 1.00 |
| - Croatia | ·· | ·· | ·· | ·· | 0.01 | 0.01 | 0.00 | 1.00 |
| Initial interview wave: |  |  |  |  |  |  |  |  |
| - Wave 1 | 0.12 | 0.12 | -0.09 | 0.93 | 0.09 | 0.09 | -0.35 | 0.72 |
| - Wave 2 | 0.29 | 0.29 | 0.29 | 0.77 | 0.22 | 0.23 | -0.92 | 0.36 |
| - Wave 4 | 0.23 | 0.23 | -0.41 | 0.68 | 0.18 | 0.19 | -0.78 | 0.43 |
| - Wave 5 | 0.25 | 0.25 | -0.50 | 0.62 | 0.23 | 0.23 | 0.09 | 0.93 |
| - Wave 6 | 0.11 | 0.11 | 0.93 | 0.35 | 0.26 | 0.26 | 1.70 | 0.09 |
| Area of living (initial wave): |  |  |  |  |  |  |  |  |
| - A big city | 0.16 | 0.16 | 0.20 | 0.85 | 0.17 | 0.17 | 0.37 | 0.71 |
| - Suburbs or a large town | 0.28 | 0.28 | -0.16 | 0.87 | 0.25 | 0.26 | -1.43 | 0.15 |
| - A small town or rural | 0.55 | 0.55 | -0.03 | 0.98 | 0.57 | 0.56 | 1.00 | 0.32 |
| - Missing | 0.01 | 0.01 | 0.19 | 0.85 | 0.01 | 0.01 | -0.16 | 0.87 |
| Place of living (initial wave): |  |  |  |  |  |  |  |  |
| - A farm house | 0.08 | 0.07 | 0.06 | 0.96 | 0.09 | 0.09 | 0.32 | 0.75 |
| - 1-2 family house free-stand./row | 0.57 | 0.57 | 0.06 | 0.95 | 0.55 | 0.55 | 0.08 | 0.94 |
| - A bldg with 3-8 flats | 0.13 | 0.13 | -0.13 | 0.90 | 0.13 | 0.13 | -0.19 | 0.85 |
| - A bldg with 9+ flats, up to 9 floors | 0.18 | 0.19 | -0.41 | 0.68 | 0.19 | 0.19 | -0.81 | 0.42 |
| - A high-rise with 9+ floors | 0.03 | 0.03 | 1.01 | 0.31 | 0.03 | 0.03 | 0.95 | 0.34 |
| - Missing | 0.01 | 0.01 | -0.15 | 0.88 | 0.01 | 0.01 | 0.89 | 0.38 |
| Number of children alive (initial wave): |  |  |  |  |  |  |  |  |
| - No children | 0.05 | 0.05 | 0.00 | 1.00 | 0.05 | 0.05 | 0.80 | 0.42 |
| - 1 child | 0.18 | 0.17 | 0.91 | 0.36 | 0.18 | 0.18 | -0.27 | 0.79 |
| - 2 children | 0.36 | 0.39 | -2.09 | 0.04 | 0.37 | 0.39 | -1.86 | 0.06 |
| - 3+ children | 0.41 | 0.39 | 1.36 | 0.17 | 0.40 | 0.38 | 1.70 | 0.09 |
| Number of grandchildren (initial wave): |  |  |  |  |  |  |  |  |
| - No grandchildren | 0.17 | 0.17 | 0.19 | 0.85 | 0.16 | 0.16 | -0.77 | 0.44 |
| - 1 grandchild | 0.09 | 0.10 | -0.68 | 0.49 | 0.09 | 0.10 | -1.45 | 0.15 |
| - 2 grandchildren | 0.17 | 0.15 | 1.65 | 0.10 | 0.17 | 0.16 | 1.62 | 0.11 |
| - 3+ grandchildren | 0.57 | 0.58 | -0.91 | 0.36 | 0.58 | 0.58 | 0.31 | 0.76 |
| Age at first birth: |  |  |  |  |  |  |  |  |
| - First child at age <25 | 0.52 | 0.52 | -0.12 | 0.91 | 0.55 | 0.53 | 1.46 | 0.14 |
| - First child at age 25+ | 0.40 | 0.40 | -0.03 | 0.98 | 0.38 | 0.39 | -1.60 | 0.11 |
| - Ever had children, age unknown | 0.04 | 0.04 | 0.15 | 0.88 | 0.03 | 0.04 | -0.76 | 0.45 |
| - Never had any children | 0.04 | 0.04 | 0.23 | 0.82 | 0.04 | 0.04 | 0.98 | 0.33 |
| Respondent's health in childhood |  |  |  |  |  |  |  |  |
| - Excellent/very good | 0.64 | 0.63 | 1.20 | 0.23 | 0.61 | 0.61 | 0.63 | 0.53 |
| - Good | 0.27 | 0.27 | -0.49 | 0.62 | 0.29 | 0.28 | 0.17 | 0.87 |
| - Fair | 0.07 | 0.07 | -1.31 | 0.19 | 0.08 | 0.08 | -0.70 | 0.48 |
| - Poor | 0.02 | 0.02 | 0.19 | 0.85 | 0.02 | 0.03 | -0.81 | 0.42 |
| - Health varied a great deal | 0.00 | 0.01 | -0.63 | 0.53 | 0.00 | 0.00 | -0.20 | 0.84 |
| Respondent had a psychiatric problem as a child | 0.01 | 0.01 | 0.27 | 0.78 | 0.01 | 0.01 | -0.92 | 0.36 |
| Partner's age (initial wave) | 73.77 | 74.04 | -1.05 | 0.29 | 74.27 | 74.42 | -0.66 | 0.51 |
| Partner's education: |  |  |  |  |  |  |  |  |
| - Primary or less | 0.38 | 0.38 | -0.27 | 0.79 | 0.36 | 0.37 | -0.34 | 0.73 |
| - Secondary and post-secondary non-tertiary | 0.45 | 0.45 | -0.03 | 0.98 | 0.46 | 0.47 | -0.64 | 0.52 |
| - Tertiary | 0.14 | 0.15 | -0.46 | 0.65 | 0.15 | 0.14 | 0.33 | 0.74 |
| - Other | 0.03 | 0.02 | 2.02 | 0.04 | 0.03 | 0.02 | 2.52 | 0.01 |
| Partner's health (initial wave): |  |  |  |  |  |  |  |  |
| - Excellent | 0.02 | 0.02 | -0.59 | 0.55 | 0.02 | 0.02 | -0.18 | 0.86 |
| - Very good | 0.08 | 0.07 | 1.94 | 0.05 | 0.06 | 0.06 | -0.65 | 0.52 |
| - Good | 0.21 | 0.21 | 0.07 | 0.94 | 0.20 | 0.20 | -1.19 | 0.23 |
| - Fair | 0.33 | 0.34 | -0.71 | 0.48 | 0.35 | 0.34 | 0.83 | 0.41 |
| - Poor | 0.35 | 0.35 | -0.27 | 0.78 | 0.38 | 0.37 | 0.45 | 0.65 |
| - Missing | 0.01 | 0.01 | 0.20 | 0.84 | 0.01 | 0.01 | 0.54 | 0.59 |
| Partner's BMI category (initial wave): |  |  |  |  |  |  |  |  |
| - Underweight | 0.01 | 0.02 | -0.59 | 0.56 | 0.02 | 0.02 | 0.55 | 0.58 |
| - Normal weight | 0.39 | 0.38 | 0.62 | 0.53 | 0.36 | 0.38 | -1.61 | 0.11 |
| - Overweight | 0.40 | 0.42 | -1.18 | 0.24 | 0.42 | 0.41 | 0.31 | 0.76 |
| - Obese | 0.18 | 0.17 | 0.57 | 0.57 | 0.18 | 0.17 | 0.96 | 0.34 |
| - Missing | 0.02 | 0.01 | 1.09 | 0.28 | 0.02 | 0.02 | 1.27 | 0.20 |
| Partner's smoking behavior (initial wave): |  |  |  |  |  |  |  |  |
| - Never smoked | 0.31 | 0.32 | -0.41 | 0.68 | 0.33 | 0.33 | 0.19 | 0.85 |
| - Smoked up to 16 years | 0.09 | 0.10 | -1.13 | 0.26 | 0.10 | 0.10 | -0.08 | 0.93 |
| - Smoked 16-30 years | 0.16 | 0.17 | -1.09 | 0.27 | 0.16 | 0.16 | -1.27 | 0.20 |
| - Smoked over 30 years | 0.41 | 0.39 | 1.51 | 0.13 | 0.38 | 0.39 | -0.42 | 0.67 |
| - Missing | 0.03 | 0.02 | 1.24 | 0.22 | 0.03 | 0.02 | 3.78 | 0.00 |
| Partner never had siblings | 0.11 | 0.10 | 0.24 | 0.81 | 0.11 | 0.10 | 0.58 | 0.56 |
| Partner's father's alive or age at death: |  |  |  |  |  |  |  |  |
| - Dead age <50 | 0.10 | 0.10 | 0.87 | 0.39 | 0.08 | 0.09 | -2.18 | 0.03 |
| - Dead age 50-60 | 0.10 | 0.10 | 0.75 | 0.45 | 0.10 | 0.09 | 0.87 | 0.38 |
| - Dead age 60-64 | 0.08 | 0.07 | 0.44 | 0.66 | 0.09 | 0.08 | 1.26 | 0.21 |
| - Dead age 65-79 | 0.37 | 0.36 | 0.42 | 0.67 | 0.38 | 0.36 | 1.43 | 0.15 |
| - Dead age 80-84 | 0.14 | 0.14 | 0.00 | 1.00 | 0.12 | 0.14 | -1.37 | 0.17 |
| - Dead age 85-89 | 0.08 | 0.09 | -1.02 | 0.31 | 0.08 | 0.09 | -0.95 | 0.34 |
| - Dead age 90+ | 0.05 | 0.06 | -1.80 | 0.07 | 0.06 | 0.06 | 0.16 | 0.87 |
| - Dead unknown age | 0.04 | 0.04 | -0.73 | 0.47 | 0.05 | 0.05 | 0.11 | 0.91 |
| - Alive | 0.02 | 0.02 | 0.11 | 0.91 | 0.02 | 0.02 | -0.10 | 0.92 |
| - Missing | 0.02 | 0.02 | 0.20 | 0.84 | 0.02 | 0.02 | 0.00 | 1.00 |
| Partner had been diagnosed before age 45 with: |  |  |  |  |  |  |  |  |
| - High blood pressure | 0.06 | 0.06 | 1.09 | 0.27 | 0.05 | 0.06 | -0.79 | 0.43 |
| - Stroke | 0.00 | 0.00 | -0.22 | 0.83 | 0.01 | 0.00 | 0.73 | 0.46 |
| - Diabetes | 0.02 | 0.02 | 0.53 | 0.59 | 0.02 | 0.02 | -0.10 | 0.92 |
| - Stomach or duodenal ulcer | 0.03 | 0.03 | -0.81 | 0.42 | 0.04 | 0.04 | 0.41 | 0.68 |
| - Hip fracture | 0.00 | 0.00 | -1.73 | 0.08 | 0.00 | 0.00 | -0.66 | 0.51 |
| - Arthritis | 0.03 | 0.03 | 0.00 | 1.00 | 0.03 | 0.03 | -0.55 | 0.59 |
| Observations | 2381 | 2381 |  |  | 3076 | 3076 |  |  |

Source: own calculations based on SHARE data.

Note: Missing categories - due to different item non-response between variables employed in the matching procedure, we used a separate, additional category to account for missing information in each case.

**Table S4 Information on specific survey questions analyzed in the paper**

| **Measure** | **Survey, wave** | **Question text** |
| --- | --- | --- |
| EURO-D depression scale (1 means having a specific symptom, 0 otherwise) | SHARE, waves 1-2, 4-7 | What are your hopes for the future?  0. Any hopes mentioned  1. No hopes mentioned |
|  |  | In the last month, have you felt that you would rather be dead?  1. Any mention of suicidal feelings or wishing to be dead  0. No such feelings |
|  |  | Do you tend to blame yourself or feel guilty about anything?  1. Obvious excessive guilt or self-blame  0. No such feelings |
|  |  | Have you had trouble sleeping recently?  1. Trouble with sleep or recent change in pattern  0. No trouble sleeping |
|  |  | In the last month, what is your interest in things?  1. Less interest than usual mentioned  0. No mention of loss of interest |
|  |  | Have you been irritable recently?  1. Yes  0. No |
|  |  | What has your appetite been like?  1. Diminution in desire for food  0. No diminution in desire for food |
|  |  | In the last month, have you had too little energy to do the things you wanted to do?  1. Yes  0. No |
|  |  | How is your concentration? For example, can you concentrate on a television programme, film or radio programme?  1. Difficulty in concentrating on entertainment  0. No such difficulty mentioned |
|  |  | Can you concentrate on something you read?  1. Difficulty in concentrating on reading  0. No such difficulty mentioned |
|  |  | What have you enjoyed doing recently?  1. Fails to mention any enjoyable activity  0. Mentions any enjoyment from activity |
|  |  | In the last month, have you cried at all?  1. Yes  0. No |
| Life satisfaction | SHARE, wave 1* | How satisfied are you with your life in general?  1. Very satisfied  2. Somewhat satisfied  3. Somewhat dissatisfied  4. Very dissatisfied |
|  | SHARE, waves 2, 4-7 | On a scale from 0 to 10 where 0 means completely dissatisfied and 10 means completely satisfied, how satisfied are you with your life? |
|  | American Time Use Survey 2012-2013 | Please imagine a ladder with steps numbered from 0 at the bottom to 10 at the top. The top of the ladder represents the best possible life for you and the bottom of the ladder represents the worst possible life for you. If the top step is 10 and the bottom step is 0, on which step of the ladder do you feel you personally stand at the present time? |
|  | French Time Use Survey 2009-2010 | All things considered, how satisfied would you say you are with your current life in general? Please rate your feelings using a scale of 1 to 10 where 1 means  “Very dissatisfied” and 10 means “Very satisfied”. |
|  | Polish Time Use Survey 2013 | How satisfied are you with your life in general?  On a scale of 5-1 (very dissatisfied – very satisfied). |
|  | United Kingdom Time Use Survey 2014-2015 | How dissatisfied or satisfied would you say you are with your life overall?  On a scale of 7-0 |
| Loneliness | SHARE, wave 6 | How much of the time do you feel lonely  1. Often  2. Some of the time  3. Hardly ever or never |

Source: Own compilation based on questionnaires from SHARE survey and American, French, Polish and United Kingdom Time Use Surveys.

Note: * These answers were translated into the 10-0 scale based on comparable sample proportions. ‘Very satisfied’: 10-9, ‘Somewhat satisfied’: 8-7, ‘Somewhat dissatisfied’: 6-4 and ‘Very dissatisfied’: 3 or less.

**Table S5 Results of the OLS regression on indicator of life satisfaction (SHARE)**

|  | **Specification 1** | | **Specification 2** | | **Specification 3** | | **Specification 4** | |
| --- | --- | --- | --- | --- | --- | --- | --- | --- |
|  | **Coefficient** | **(s.e.)** | **Coefficient** | **(s.e.)** | **Coefficient** | **(s.e.)** | **Coefficient** | **(s.e.)** |
| Widow | -0.1027 | (0.0270) | -0.1089 | (0.0275) | -0.1076 | (0.0275) | -0.1043 | (0.0276) |
| Age (ref: 70-74) |  |  |  |  |  |  |  |  |
| - 75-79 |  |  | 0.0308 | (0.0189) | 0.0282 | (0.0189) | 0.0305 | (0.0189) |
| - 80-84 |  |  | 0.0847 | (0.0245) | 0.0819 | (0.0245) | 0.0831 | (0.0245) |
| - 85+ |  |  | 0.0639 | (0.0395) | 0.0621 | (0.0396) | 0.0654 | (0.0396) |
| Education (ref: Primary or less) |  |  |  |  |  |  |  |  |
| - Secondary and post-secondary non-tertiary |  |  | 0.0356 | (0.0190) | 0.0376 | (0.0194) | 0.0363 | (0.0194) |
| - Tertiary |  |  | 0.0285 | (0.0260) | 0.0287 | (0.0266) | 0.0212 | (0.0267) |
| - Other |  |  | 0.0828 | (0.0804) | 0.0809 | (0.0805) | 0.0795 | (0.0803) |
| Area of living (ref: A rural area) |  |  |  |  |  |  |  |  |
| - A big city |  |  | -0.0136 | (0.0270) | -0.0057 | (0.0273) | -0.0067 | (0.0273) |
| - Suburbs |  |  | 0.0311 | (0.0311) | 0.0373 | (0.0313) | 0.0359 | (0.0312) |
| - A large town |  |  | 0.0006 | (0.0255) | 0.0035 | (0.0258) | 0.0054 | (0.0258) |
| - A small town |  |  | 0.0566 | (0.0215) | 0.0561 | (0.0216) | 0.0581 | (0.0216) |
| House ownership (ref: Owner) |  |  |  |  |  |  |  |  |
| - Member of a cooperative |  |  | 0.0981 | (0.0618) | 0.0944 | (0.0619) | 0.0874 | (0.0618) |
| - Tenant |  |  | 0.0157 | (0.0301) | 0.0146 | (0.0302) | 0.0118 | (0.0302) |
| - Subtenant |  |  | 0.1602 | (0.1441) | 0.1639 | (0.1442) | 0.1636 | (0.1440) |
| - Rent free |  |  | 0.0438 | (0.0320) | 0.0419 | (0.0322) | 0.0406 | (0.0321) |
| Health (ref: Poor) |  |  |  |  |  |  |  |  |
| - Excellent |  |  | 0.4442 | (0.0483) | 0.4426 | (0.0486) | 0.4275 | (0.0486) |
| - Very good |  |  | 0.4279 | (0.0338) | 0.4234 | (0.0339) | 0.4165 | (0.0339) |
| - Good |  |  | 0.3340 | (0.0274) | 0.3327 | (0.0275) | 0.3304 | (0.0275) |
| - Fair |  |  | 0.1657 | (0.0272) | 0.1682 | (0.0272) | 0.1665 | (0.0272) |
| Number of children alive (ref: No children) |  |  |  |  |  |  |  |  |
| - 1 child |  |  |  |  | -0.0287 | (0.0487) | -0.0206 | (0.0498) |
| - 2 children |  |  |  |  | -0.0090 | (0.0458) | 0.0001 | (0.0471) |
| - 3+ children |  |  |  |  | 0.0208 | (0.0453) | 0.0297 | (0.0467) |
| Distance and frequency of contact with children (ref: Same household) |  |  |  |  |  |  |  |  |
| - Same building or less than 1km and contact every day |  |  |  |  | 0.0271 | (0.0280) | 0.0217 | (0.0280) |
| - Between 1-5km and contact every day |  |  |  |  | 0.0134 | (0.0380) | 0.0085 | (0.0380) |
| - Less than 5km and contact less often |  |  |  |  | 0.0359 | (0.0283) | 0.0385 | (0.0283) |
| - Further than 5km and contact every day |  |  |  |  | -0.0229 | (0.0373) | -0.0252 | (0.0373) |
| - Further than 5km and contact more than once a week |  |  |  |  | 0.0436 | (0.0318) | 0.0421 | (0.0317) |
| - Further than 5km and contact less often |  |  |  |  | -0.0090 | (0.0339) | -0.0099 | (0.0342) |
| Characteristics of the social network (SN, dummies)*: |  |  |  |  |  |  |  |  |
| Empty SN |  |  |  |  |  |  | 0.0821 | (0.0713) |
| Partner with whom one feels close in SN |  |  |  |  |  |  | 0.0517 | (0.0188) |
| Child with whom one feels close in SN |  |  |  |  |  |  | -0.0062 | (0.0177) |
| Friend with whom one feels close in SN |  |  |  |  |  |  | 0.0425 | (0.0211) |
| Satisfied with SN |  |  |  |  |  |  | 0.0457 | (0.0169) |
| Constant | 0.7403 | (0.0447) | 0.2783 | (0.0478) | 0.2614 | (0.0614) | 0.1904 | (0.0637) |
| *N* | 3056 |  | 3056 |  | 3056 |  | 3056 |  |
| *R*^2^ | 0.1092 |  | 0.1085 |  | 0.1120 |  | 0.1176 |  |

Source: own calculations based on SHARE data.

Notes: ‘Satisfied with life’ = 1 if >7 on a 10-0 scale. In addition to variables listed in the Table, Specifications 1-4 control for country, and Specifications 2-4 for month of interview. * SN – social network.

**Table S6 Results of the OLS regression on indicator of feeling lonely (SHARE)**

|  | **Specification 1** | | **Specification 2** | | **Specification 3** | | **Specification 4** | |
| --- | --- | --- | --- | --- | --- | --- | --- | --- |
|  | **Coeff.** | **(s.e.)** | **Coeff.** | **(s.e.)** | **Coeff.** | **(s.e.)** | **Coeff.** | **(s.e.)** |
| Widow | 0.3766 | (0.0245) | 0.3648 | (0.0249) | 0.3631 | (0.0249) | 0.3636 | (0.0250) |
| Age (ref: 70-74) |  |  |  |  |  |  |  |  |
| - 75-79 |  |  | 0.0240 | (0.0171) | 0.0242 | (0.0171) | 0.0227 | (0.0171) |
| - 80-84 |  |  | -0.0068 | (0.0221) | -0.0081 | (0.0222) | -0.0076 | (0.0222) |
| - 85+ |  |  | 0.0241 | (0.0357) | 0.0179 | (0.0358) | 0.0164 | (0.0358) |
| Education (ref: Primary or less) |  |  |  |  |  |  |  |  |
| - Secondary and post-secondary non-tertiary |  |  | -0.1125 | (0.0171) | -0.1154 | (0.0175) | -0.1151 | (0.0176) |
| - Tertiary |  |  | -0.1211 | (0.0235) | -0.1235 | (0.0240) | -0.1218 | (0.0242) |
| - Other |  |  | 0.0026 | (0.0727) | 0.0035 | (0.0727) | 0.0083 | (0.0727) |
| Area of living (ref: A rural area) |  |  |  |  |  |  |  |  |
| - A big city |  |  | 0.0379 | (0.0244) | 0.0361 | (0.0247) | 0.0354 | (0.0247) |
| - Suburbs |  |  | 0.0048 | (0.0282) | 0.0016 | (0.0283) | 0.0033 | (0.0283) |
| - A large town |  |  | 0.0032 | (0.0231) | 0.0026 | (0.0233) | 0.0017 | (0.0233) |
| - A small town |  |  | -0.0189 | (0.0195) | -0.0170 | (0.0195) | -0.0169 | (0.0196) |
| House ownership (ref: Owner) |  |  |  |  |  |  |  |  |
| - Member of a cooperative |  |  | -0.0901 | (0.0559) | -0.0985 | (0.0560) | -0.0933 | (0.0559) |
| - Tenant |  |  | -0.0362 | (0.0272) | -0.0409 | (0.0273) | -0.0405 | (0.0273) |
| - Subtenant |  |  | 0.0347 | (0.1303) | 0.0258 | (0.1304) | 0.0176 | (0.1303) |
| - Rent free |  |  | 0.0334 | (0.0289) | 0.0430 | (0.0291) | 0.0448 | (0.0291) |
| Health (ref: Poor) |  |  |  |  |  |  |  |  |
| - Excellent |  |  | -0.2648 | (0.0437) | -0.2690 | (0.0439) | -0.2576 | (0.0440) |
| - Very good |  |  | -0.1702 | (0.0306) | -0.1742 | (0.0307) | -0.1706 | (0.0307) |
| - Good |  |  | -0.1527 | (0.0248) | -0.1568 | (0.0249) | -0.1567 | (0.0249) |
| - Fair |  |  | -0.0649 | (0.0246) | -0.0660 | (0.0246) | -0.0658 | (0.0246) |
| Number of children alive (ref: No children) |  |  |  |  |  |  |  |  |
| - 1 child |  |  |  |  | -0.0283 | (0.0440) | -0.0297 | (0.0451) |
| - 2 children |  |  |  |  | -0.0411 | (0.0414) | -0.0416 | (0.0426) |
| - 3+ children |  |  |  |  | -0.0276 | (0.0410) | -0.0284 | (0.0423) |
| Distance and frequency of contact with children (ref: Same household) |  |  |  |  |  |  |  |  |
| - Same building or less than 1km and contact every day |  |  |  |  | -0.0588 | (0.0253) | -0.0566 | (0.0253) |
| - Between 1-5km and contact every day |  |  |  |  | -0.0350 | (0.0344) | -0.0328 | (0.0344) |
| - Less than 5km and contact less often |  |  |  |  | 0.0039 | (0.0256) | -0.0001 | (0.0256) |
| - Further than 5km and contact every day |  |  |  |  | 0.0153 | (0.0337) | 0.0145 | (0.0338) |
| - Further than 5km and contact more than once a week |  |  |  |  | -0.0331 | (0.0287) | -0.0341 | (0.0287) |
| - Further than 5km and contact less often |  |  |  |  | 0.0147 | (0.0307) | 0.0121 | (0.0310) |
| Characteristics of the social network (SN, dummies)*: |  |  |  |  |  |  |  |  |
| Empty SN |  |  |  |  |  |  | 0.0328 | (0.0645) |
| Partner with whom one feels close in SN |  |  |  |  |  |  | -0.0277 | (0.0170) |
| Child with whom one feels close in SN |  |  |  |  |  |  | -0.0013 | (0.0160) |
| Friend with whom one feels close in SN |  |  |  |  |  |  | 0.0015 | (0.0191) |
| Satisfied with SN |  |  |  |  |  |  | -0.0439 | (0.0153) |
| Constant | 0.0992 | (0.0405) | 0.3493 | (0.0432) | 0.3997 | (0.0555) | 0.4476 | (0.0576) |
| *N* | 3056 |  | 3056 |  | 3056 |  | 3056 |  |
| *R*^2^ | 0.1266 |  | 0.1296 |  | 0.1341 |  | 0.1380 |  |

Source: own calculations based on SHARE data.

Notes: ‘Feeling lonely’ if indicated experiencing loneliness often or some of the time. In addition to variables listed in the Table, Specifications 1-4 control for country, and Specifications 2-4 for month of interview. * SN – social network.

**Table S7 Life satisfaction, time use and widowhood in Poland**

| **Dependent variable: indicator for life satisfaction higher than 3 on the 5-1 scale** | **Specification 1** | | **Specification 2** | | **Specification 3** | | **Specification 4** | |
| --- | --- | --- | --- | --- | --- | --- | --- | --- |
|  | **Coefficient** | **(s.e.)** | **Coefficient** | **(s.e.)** | **Coefficient** | **(s.e.)** | **Coefficient** | **(s.e.)** |
|  |  |  |  |  |  |  |  |  |
| Widow | -0.1355 | (0.0184) | -0.0672 | (0.0213) | -0.0641 | (0.0214) | -0.0174 | (0.0375) |
| Age: |  |  |  |  |  |  |  |  |
| - 70-74 (omitted group) |  |  |  |  |  |  |  |  |
| - 75-79 |  |  | -0.0113 | (0.0227) | -0.0019 | (0.0226) | -0.0022 | (0.0226) |
| - 80-84 |  |  | 0.0040 | (0.0273) | 0.0230 | (0.0272) | 0.0235 | (0.0271) |
| - 85+ |  |  | -0.0182 | (0.0366) | 0.0105 | (0.0367) | 0.0119 | (0.0367) |
| Time spent (hours/day) on: |  |  |  |  |  |  |  |  |
| - Home production |  |  |  |  | -0.0020 | (0.0044) | -0.0026 | (0.0045) |
| - Sleep |  |  |  |  | -0.0295 | (0.0066) | -0.0352 | (0.0073) |
| - Other personal |  |  |  |  | -0.0122 | (0.0081) | -0.0125 | (0.0081) |
| - TV watching |  |  |  |  | -0.0147 | (0.0053) | -0.0159 | (0.0054) |
| - Other leisure (omitted category) |  |  |  |  |  |  |  |  |
| Time spent (hours/day): |  |  |  |  |  |  |  |  |
| - Alone |  |  |  |  |  |  | -0.0051 | (0.0038) |
| - With others (non-spouse) |  |  |  |  |  |  | -0.0081 | (0.0045) |
|  |  |  |  |  |  |  |  |  |
| Constant: | 0.8120 | (0.0138) | 0.4698 | (0.0736) | 0.8752 | (0.1196) | 0.9734 | (0.1316) |
|  |  |  |  |  |  |  |  |  |
| Number of observations (diaries) | 5291 | | 5291 | | 5291 | | 5291 | |
| R-squared (adj.) | 0.0186 | | 0.0571 | | 0.0671 | | 0.0680 | |
|  |  |  |  |  |  |  |  |  |

Source: own calculations based on Polish Time Use Survey 2013.

Note: Non-working widows. In addition to variables listed in the Table, Specifications 2-4 control for: when interview conducted (month and day of the week), region, size of city, immigrant status, education, equivalized income, and equivalized income squared. Time measured in hours per day. If available, diaries from two different days included per person. Standard errors clustered at individual level.

**Table S8 Life satisfaction, time use and widowhood in the U.S.**

| **Dependent variable: indicator for life satisfaction higher than 7 on the 10-0 scale** | **Specification 1** | | **Specification 2** | | **Specification 3** | | **Specification 4** | |
| --- | --- | --- | --- | --- | --- | --- | --- | --- |
|  | **Coefficient** | **(s.e.)** | **Coefficient** | **(s.e.)** | **Coefficient** | **(s.e.)** | **Coefficient** | **(s.e.)** |
|  |  |  |  |  |  |  |  |  |
| Widow | -0.0817 | (0.0314) | -0.0793 | (0.0325) | -0.0759 | (0.0331) | 0.0309 | (0.0455) |
| Age: |  |  |  |  |  |  |  |  |
| - 70-74 (omitted group) |  |  |  |  |  |  |  |  |
| - 75-79 |  |  | 0.0205 | (0.0397) | 0.0175 | (0.0397) | 0.0163 | (0.0395) |
| - 80-84 |  |  | 0.0007 | (0.0416) | -0.0051 | (0.0417) | -0.0041 | (0.0416) |
| - 85+ |  |  | 0.0340 | (0.0499) | 0.0219 | (0.0501) | 0.0180 | (0.0500) |
| White (non-Hispanic) |  |  | 0.0148 | (0.0426) | 0.0119 | (0.0427) | 0.0051 | (0.0425) |
| Immigrant |  |  | 0.0851 | (0.0575) | 0.0907 | (0.0576) | 0.0815 | (0.0573) |
| Time spent (hours/day) on: |  |  |  |  |  |  |  |  |
| - Home production |  |  |  |  | -0.0022 | (0.0060) | 0.0004 | (0.0061) |
| - Sleep |  |  |  |  | -0.0006 | (0.0078) | -0.0110 | (0.0084) |
| - Other personal |  |  |  |  | 0.0302 | (0.0107) | 0.0221 | (0.0110) |
| - TV watching |  |  |  |  | 0.0008 | (0.0058) | 0.0008 | (0.0060) |
| - Other leisure (omitted category) |  |  |  | |  | |  | |
| Time spent (hours/day): |  |  |  |  |  |  |  |  |
| - Alone |  |  |  |  |  |  | -0.0157 | (0.0045) |
| - With others (non-spouse) |  |  |  |  |  |  | -0.0142 | (0.0064) |
|  |  |  |  |  |  |  |  |  |
| Constant: | 0.7169 | (0.0226) | 0.8383 | (0.1043) | 0.7863 | (0.1401) | 0.9779 | (0.1503) |
|  |  |  |  |  |  |  |  |  |
| Number of observations | 888 | | 888 | | 888 | | 888 | |
| R-squared (adj.) | 0.0065 | | 0.1196 | | 0.1253 | | 0.1359 | |
|  |  |  |  |  |  |  |  |  |

Source: own calculations based on American Time Use Survey 2012-2013.

Note: Non-working widows. In addition to variables listed in the Table, Specifications 2-4 control for: when interview conducted (year, month and day of the week), Census region, rural location, immigrant status, race/ethnicity, education, general health, and income. Time measured in hours per day. Life satisfaction in ATUS was collected only in years 2012-2013.

**Table S9 Life satisfaction, time use and widowhood in the U.K.**

| **Dependent variable: indicator for life satisfaction higher than 5 on the 7-1 scale** | **Specification 1** | | **Specification 2** | | **Specification 3** | | **Specification 4** | |
| --- | --- | --- | --- | --- | --- | --- | --- | --- |
|  | **Coefficient** | **(s.e.)** | **Coefficient** | **(s.e.)** | **Coefficient** | **(s.e.)** | **Coefficient** | **(s.e.)** |
|  |  |  |  |  |  |  |  |  |
| Widow | -0.1946 | (0.0544) | -0.1932 | (0.0672) | -0.2139 | (0.0688) | 0.0295 | (0.0926) |
| Age: |  |  |  |  |  |  |  |  |
| - 70-74 (omitted group) |  |  |  |  |  |  |  |  |
| - 75-79 |  |  | 0.0753 | (0.1112) | 0.0602 | (0.1125) | 0.1158 | (0.1103) |
| - 80-84 |  |  | 0.0608 | (0.1116) | 0.0564 | (0.1132) | 0.0382 | (0.1096) |
| - 85+ |  |  | 0.0263 | (0.1159) | 0.0384 | (0.1170) | 0.0512 | (0.1130) |
| Time spent (hours/day) on: |  |  |  |  |  |  |  |  |
| - Home production |  |  |  |  | -0.0058 | (0.0154) | -0.0108 | (0.0151) |
| - Sleep |  |  |  |  | -0.0279 | (0.0193) | -0.0353 | (0.0188) |
| - Other personal |  |  |  |  | -0.0152 | (0.0236) | -0.0153 | (0.0230) |
| - TV watching |  |  |  |  | -0.0192 | (0.0152) | -0.0253 | (0.0147) |
| - Other leisure (omitted category) |  |  |  |  |  |  |  |  |
| Time spent (hours/day): |  |  |  |  |  |  |  |  |
| - Alone |  |  |  |  |  |  | -0.0150 | (0.0050) |
| - With others (non-spouse) |  |  |  |  |  |  | -0.0199 | (0.0046) |
|  |  |  |  |  |  |  |  |  |
| Constant: | 0.7882 | (0.0371) | 0.0213 | (0.3881) | 0.3772 | (0.4758) | 0.4724 | (0.4640) |
|  |  |  |  |  |  |  |  |  |
| Number of observations | 276 | | 276 | | 276 | | 276 | |
| R-squared (adj.) | 0.0412 | | 0.1096 | | 0.1075 | | 0.1701 | |
|  |  |  |  |  |  |  |  |  |

Source: own calculations based on United Kingdom Time Use Survey 2014-2015.

Note: In addition to variables listed in the Table, Specifications 2-4 control for: month of interview, education, and income. Time measured in hours per day. Life satisfaction in UKTUS was collected only for a subsample of participants.

**Table S10 Life satisfaction, time use and widowhood in France**

| **Dependent variable: indicator for life satisfaction higher than 6 on the 10-0 scale** | **Specification 1** | | **Specification 2** | | **Specification 3** | | **Specification 4** | |
| --- | --- | --- | --- | --- | --- | --- | --- | --- |
|  | **Coefficient** | **(s.e.)** | **Coefficient** | **(s.e.)** | **Coefficient** | **(s.e.)** | **Coefficient** | **(s.e.)** |
|  |  |  |  |  |  |  |  |  |
| Widow | -0.2677 | (0.0986) | -0.2828 | (0.1215) | -0.2464 | (0.1237) | -0.2137 | (0.2147) |
| Age: |  |  |  |  |  |  |  |  |
| - 70-74 (omitted group) |  |  |  |  |  |  |  |  |
| - 75-79 |  |  | 0.0986 | (0.1194) | 0.1189 | (0.1175) | 0.1118 | (0.1175) |
| - 80-84 |  |  | 0.1692 | (0.1316) | 0.2112 | (0.1365) | 0.1974 | (0.1389) |
| - 85+ |  |  | -0.0584 | (0.1400) | -0.0291 | (0.1412) | -0.0338 | (0.1352) |
| Time spent (hours/day) on: |  |  |  |  |  |  |  |  |
| - Home production |  |  |  |  | -0.0264 | (0.0177) | -0.0277 | (0.0183) |
| - Sleep |  |  |  |  | 0.0056 | (0.0240) | 0.0014 | (0.0290) |
| - Other personal |  |  |  |  | -0.0067 | (0.0241) | -0.0060 | (0.0242) |
| - TV watching |  |  |  |  | -0.0594 | (0.0227) | -0.0664 | (0.0259) |
| - Other leisure (omitted category) |  |  |  |  |  |  |  |  |
| Time spent (hours/day): |  |  |  |  |  |  |  |  |
| - Alone |  |  |  |  |  |  | -0.0009 | (0.0157) |
| - With others (non-spouse) |  |  |  |  |  |  | -0.0120 | (0.0223) |
|  |  |  |  |  |  |  |  |  |
| Constant: | 0.8419 | (0.0505) | 0.2374 | (0.6454) | 0.7988 | (0.7043) | 0.8453 | (0.7394) |
|  |  |  |  |  |  |  |  |  |
| Number of observations (diaries) | 206 | | 206 | | 206 | | 206 | |
| R-squared (adj.) | 0.0864 | | 0.5735 | | 0.6028 | | 0.6044 | |
|  |  |  |  |  |  |  |  |  |

Source: own calculations based on French Time Use Survey 2009-2010.

Note: Non-working widows. In addition to variables listed in the Table, Specifications 2-4 control for: when interview conducted (month and day of the week), region, size of city, education, equivalized income, equivalized income squared, general health, and type of home ownership. Time measured in hours per day. If available, diaries from two different days included per person. Standard errors clustered at individual level. Life satisfaction in French Time Use Survey was collected only for a subsample of participants.
